# Purine biosynthesis in archaea: variations on a theme

**DOI:** 10.1186/1745-6150-6-63

**Published:** 2011-12-14

**Authors:** Anne M Brown, Samantha L Hoopes, Robert H White, Catherine A Sarisky

**Affiliations:** 1 Department of Chemistry, Roanoke College, Salem, VA 24153, USA; 2 Department of Biochemistry, Virginia Polytechnic Institute and State University, Blacksburg, VA 24061-0308, USA; 3 Department of Cell and Molecular Physiology, University of North Carolina at Chapel Hill, School of Medicine, Chapel Hill, NC 27599, USA

## Abstract

**Background:**

The ability to perform *de novo *biosynthesis of purines is present in organisms in all three domains of life, reflecting the essentiality of these molecules to life. Although the pathway is quite similar in eukaryotes and bacteria, the archaeal pathway is more variable. A careful manual curation of genes in this pathway demonstrates the value of manual curation in archaea, even in pathways that have been well-studied in other domains.

**Results:**

We searched the Integrated Microbial Genome system (IMG) for the 17 distinct genes involved in the 11 steps of *de novo *purine biosynthesis in 65 sequenced archaea, finding 738 predicted proteins with sequence similarity to known purine biosynthesis enzymes. Each sequence was manually inspected for the presence of active site residues and other residues known or suspected to be required for function.

Many apparently purine-biosynthesizing archaea lack evidence for a single enzyme, either glycinamide ribonucleotide formyltransferase or inosine monophosphate cyclohydrolase, suggesting that there are at least two more gene variants in the purine biosynthetic pathway to discover. Variations in domain arrangement of formylglycinamidine ribonucleotide synthetase and substantial problems in aminoimidazole carboxamide ribonucleotide formyltransferase and inosine monophosphate cyclohydrolase assignments were also identified.

Manual curation revealed some overly specific annotations in the IMG gene product name, with predicted proteins without essential active site residues assigned product names implying enzymatic activity (21 proteins, 2.8% of proteins inspected) or Enzyme Commission (E. C.) numbers (57 proteins, 7.7%). There were also 57 proteins (7.7%) assigned overly generic names and 78 proteins (10.6%) without E.C. numbers as part of the assigned name when a specific enzyme name and E. C. number were well-justified.

**Conclusions:**

The patchy distribution of purine biosynthetic genes in archaea is consistent with a pathway that has been shaped by horizontal gene transfer, duplication, and gene loss. Our results indicate that manual curation can improve upon automated annotation for a small number of automatically-annotated proteins and can reveal a need to identify further pathway components even in well-studied pathways.

**Reviewers:**

This article was reviewed by Dr. Céline Brochier-Armanet, Dr Kira S Makarova (nominated by Dr. Eugene Koonin), and Dr. Michael Galperin.

## Background

Purines are key components of all living cells on earth, required for energy metabolism and biosynthesis of RNA and DNA. Purine biosynthesis pathways were first described in the 1950's and 1960's [1-3] and represented a central force in the development of the field of biochemistry. For decades, the story of purine biosynthesis seemed mostly complete, with only a few new enzymes added to the pathway [4-6]. However, with increased study of archaea and the availability of archaeal genomes, it became clear that the purine biosynthesis pathway in many archaea included several unique enzymes [7,8].

**Figure 1:**
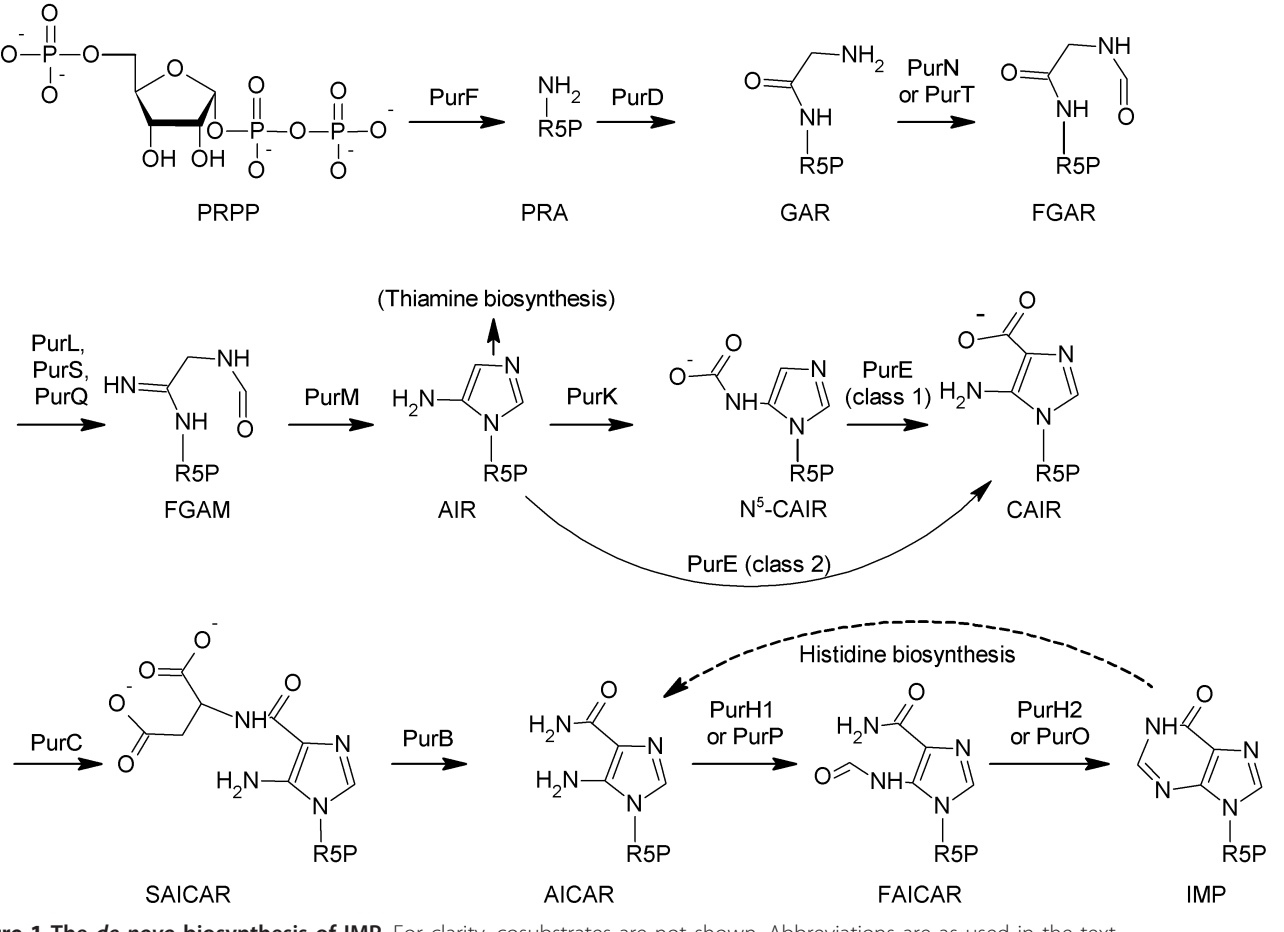
**The *de novo *biosynthesis of IMP**. For clarity, cosubstrates are not shown. Abbreviations are as used in the text.

The accepted purine biosynthesis pathway (with known variations) is shown in Figure 1. There is complete conservation of the intermediates of purine biosynthesis from phosphoribosyl pyrophosphate (PRPP) to 5-phospho-β-D-ribosylamine (PRA), with the exception of N^5^-CAIR (N^5^-carboxyaminoimidazole ribonucleotide), which is bypassed in eukaryotes. The enzymes catalyzing each step, however, are more variable, with four common nonhomologous enzyme substitutions known across the three domains. Intriguingly, the archaea manifest all four of the known nonhomologous substitutions in this pathway, with evidence (discussed in this paper) for an additional two substitutions still to be identified.

The *de novo *purine biosynthesis pathway from PRPP to IMP includes one branch, to thiamine biosynthesis, and one alternate input, from histidine biosynthesis. Thiamine biosynthesis uses AIR (aminoimidazole ribotide) as a substrate. Thus, the first four enzymatic steps of the pathway are not truly committed to purine biosynthesis, but serve both purine biosynthesis and thiamine biosynthesis. The interaction between the histidine pathway and purine biosynthesis is less easily diagrammed. ATP (an end-product of purine biosynthesis or obtained by salvage) reacts with PRPP, followed by a ring-opening reaction that opens the purine ring, leading to production of aminoimidazole carboxamide ribonucleotide (AICAR), which can be formylated and then cyclized to re-form the purine ring. Thus, provided that the final two steps of the *de novo *purine biosynthesis pathway are functional, histidine biosynthesis will result in no net loss or gain in purines. Due to the desirability of reclaiming the AICAR, we expect to find at least the final two steps of the *de novo *purine pathway present whenever the histidine biosynthesis pathway is present.

The availability of full genome sequence data for numerous archaeal species allows the comparative study of the enzymes involved in *de novo *purine biosynthesis. We used the Integrated Microbial Genome system (IMG) to curate open reading frames (ORFs) on the basis of high similarity to an experimentally characterized gene or enzyme in another species (ideally as a bidirectional best hit [9]). The presence of nearby gene candidates for the same biochemical pathway supported the annotations through "guilt by association" [10]. Although several metabolic pathway reconstructions are available elsewhere [11,12], a genomic analysis of the purine biosynthesis pathway of the archaea in the depth and breadth presented here is not otherwise available. Below, we describe our findings upon inspection of each genome and discuss these findings in the context of previous experimental data.

The ever-increasing pace of genome sequencing means that genomes will increasingly be annotated by automated or semi-automated processes. This paper serves as a case study of the errors possible in the largely automated gene annotations present in IMG. As purine biosynthesis is relatively well-understood, our findings represent a best-case scenario, with increased likelihood of problem annotations in pathways that have been less well-studied.

## Results and Discussion

**Figure 2:**
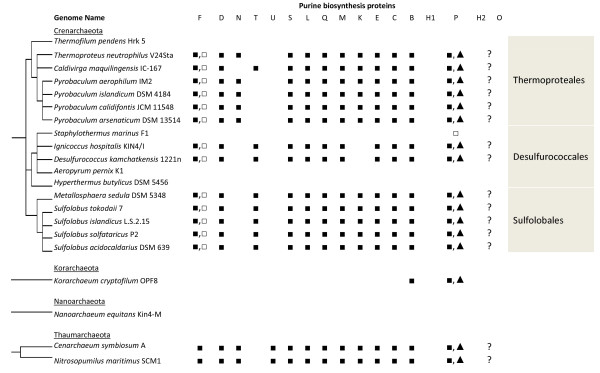
**The presence of genes for the purine biosynthesis pathway in Crenarchaeota, Nanoarchaea, Thaumarchaeota, and Korarchaea**. A schematic, taxonomy-based phylogenetic tree is provided, along with order names within the Crenarchaota. The typically bi-functional PurH protein appears twice, as PurH1 (the C-terminal AICAR formyltransferase domain) and PurH2 (the N-terminal IMP cyclohydrolase domain). Additional File 1 contains gene locus tags for each candidate gene. Symbols used: ■ denotes a gene that is a good match. □ denotes a match with some problems, as described in more detail in the text. ▲ is used to represent a cluster II PurP protein. [■] indicates that the expected gene is split into two adjacent loci. ■- ■ denotes a protein with a domain duplication. Where a "?" appears, a gene is necessary for an otherwise complete purine biosynthesis pathway to be functional, but no gene candidate could be identified with the data available.

**Figure 3:**
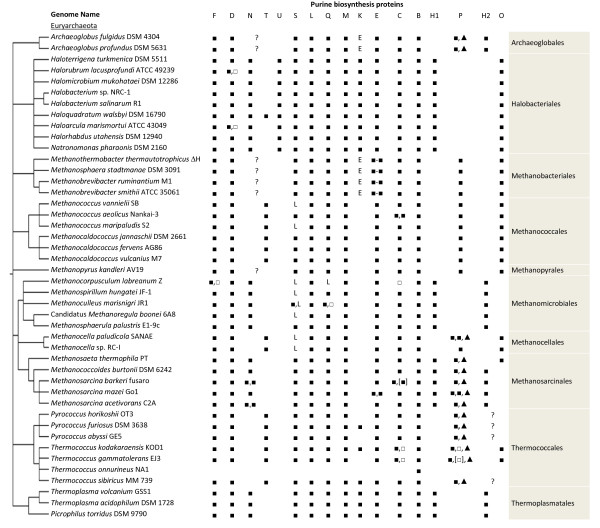
**The presence of genes for the purine biosynthesis pathway in Euryarchaeota**. Symbols are as in Figure 2. E indicates that the expected activity can be provided by the PurE protein (see text). L indicates that the expected protein sequence is encoded as part of the PurL protein (see text).

We identified a complete or nearly complete set of the genes necessary for purine biosynthesis in 58 of the 65 species studied. However, 25 of these suspected purine biosynthesizing species lacked a gene candidate for either glycinamide ribonucleotide (GAR) formyltransferase or inosine 5'-monophosphate (IMP) cyclohydrolase. As each of these 25 species lacked just one gene in the pathway, we believe that these species do synthesize purines, and that further work will reveal novel pathway variants. The gene candidates for each of the purine biosynthetic steps are listed in Additional File 1, with a summary of findings in Figures 2 and 3.

We identified at least one organism with apparent redundancy (either two similar copies of the same gene or two different genes for the same step) in five out of eleven steps in the pathway. Redundancy was especially common in the AICAR formyltransferase assignments. Only in the case of the *purB *and *purM *genes could we assign exactly one gene from each purine biosynthesizing organism to the necessary function. Specific findings for each biosynthetic step are discussed below.

### Organisms that do not synthesize purines

Our analysis revealed seven archaea with entirely absent or almost entirely absent genes encoding purine biosynthesis enzymes. On the basis of the genomic data, these organisms are incapable of biosynthesis of purines and must rely on environmental sources coupled with salvage pathways to meet their purine requirements. The non-purine synthesizing organisms identified are: *Aeropyrum pernix K1, Korarchaeum cryptofilum OPF8, Hyperthermus butylicus DSM 5456, Nanoarchaeum equitans Kin4-M, Thermococcus onnurineus NA1, Staphylothermus marinus F1*, and *Thermofilum pendens Hrk 5*. Based on the distribution of these organisms throughout the archaeal domain, the loss of purine biosynthesis genes has occurred multiple times. These organisms entirely lack the genes required for *de novo *purine biosynthesis enzymes, with only minor exceptions, described below.

Where available, the growth conditions of these non-purine biosynthesizing organisms are consistent with a requirement for purines. *Aeropyrum pernix *requires adenine in its growth medium, consistent with an inability to synthesize purines *de novo *[13]. *Korarchaeum cryptofilum *has not been grown in pure culture [14]. *Hyperthermus butylicus *[15] and *Staphylothermus marinus *[16] (both sulfur-reducing peptide fermenters) grow with tryptone or peptone in the growth medium (respectively), either of which would provide a purine source. Similarly, *Thermofilum pendens*, believed to be adapted to growth in nutrient-rich environments, requires tryptone or yeast extract for culture [17]. The parasitic *Nanoarchaeum equitans *must rely on *Ignicoccus hospitalis *to supply its purine requirement [18].

In *T. onnurineus*, which grows in rich media that could meet the organism's apparent purine requirement [19], only a *purB*-like gene is present, probably involved in purine interconversion. (We also found the purine interconversion genes *guaA, guaB*, and *purA*, not shown.) Although Lee and coworkers have proposed that *T. onnurineus *obtains purines through its histidine biosynthesis pathway [19], *de novo *histidine biosynthesis provides no net creation of purines due to use of the purine ring of ATP as a substrate, as described above. Additionally, there is no genomic evidence of a PurP or PurH enzyme that could convert the aminoimidazole carboxamide ribonucleotide (AICAR) produced by histidine biosynthesis back into a purine. If *T. onnurineus *truly lacks a means to convert the AICAR produced by histidine biosynthesis back to a purine, biosynthesis of histidine will in fact result in a net loss of purines and accumulation of AICAR in this organism. Thus, we suspect that *T. onnurineus *is auxotrophic for purines, with higher need for purines (and accumulation of AICAR) in the absence of environmental histidine.

In the case of *K. cryptofilum*, which has been enriched only in complex media (in which purines would be present) and is thought to use peptides as an energy source [14], we identified a pair of *purP*-like genes, along with *purB*. As PurB functions in purine interconversion as well as *de novo *biosynthesis, we do not consider its presence indicative of *de novo *purine synthesis. The role of PurP-like enzymes, however, is not entirely clear in this organism, as will be discussed in further detail below.

### Glutamine phosphoribosylpyrophosphate amidotransferase (EC 2.4.2.14, GPATase, PurF)

The *purF *gene product, glutamine phosphoribosylpyrophosphate amidotransferase (GPATase, PurF), catalyzes the first step in biosynthesis of purines, conversion of phosphoribosyl pyrophosphate (PRPP) to 5-phospho-β-D-ribosylamine (PRA). The *Escherichia coli *PurF enzyme has also been demonstrated to have low-level promiscuity for phosphoribosylanthranilate isomerase activity (normally encoded by *trpF*), as demonstrated by complementation studies [20].

A conserved N-terminal cysteine residue forms a catalytic triad with histidine and aspartate in the glutamine amidotransfer domain. In *Bacillus subtilis*, this is accomplished by cleavage of a short peptide from the N-terminus [21]. In *E. coli*, the cysteine is N-terminal after removal of the initial methionine. In *E. coli*, the N-terminal cysteine is required for use of glutamine as the nitrogen source, with no detectable activity when the cysteine is replaced with alanine or serine, although ammonia can still serve as the nitrogen source [22]. We thus expect any archaeal *purF *gene should encode a cysteine at or near the N-terminus if glutamine is the nitrogen source.

We identified a gene encoding a protein similar to PurF in all of the purine biosynthesizing archaeal species studied, as shown in Figures 2 and 3. Out of the Euryarchaeota studied, only *Methanocorpusculum labreanum*'s genome includes two candidate sequences, Mlab_0202 and Mlab_0150. As Mlab_0150's predicted protein has a serine in place of the active site cysteine, we suspect that Mlab_0150 does not encode a glutamine phosphoribosylpyrophosphate amidotransferase, and that Mlab_0202 is the actual *purF *gene. Mlab_150 appears to be a relatively recent duplication of Mlab_0202, as it is most similar to Mlab_0202 and the putative *purF*s in closely related species.

**Figure 4:**
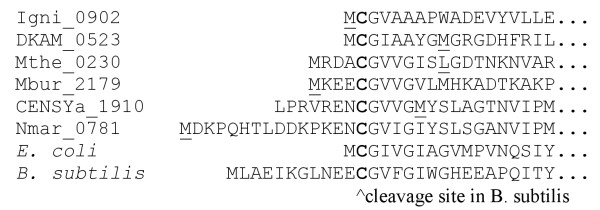
**N-terminal sequences from selected PurF candidates**. Start sites in IMG are underlined, with protein sequences for our proposed earlier start sites shown for DKAM_0523, Mthe_0230, and CENSYa_1910. The cysteine nucleophile is in bold.

The most promising PurF candidates in *Methanosaeta thermophila, Desulfurococcus kamchatkensis*, and *Cenarchaeum symbiosum *initially appeared to be lacking the active site cysteine, but when we inspected the 5' DNA sequence, we found that a cysteine was present if we proposed an earlier translation initiation site, as shown in Figure 4. Some archaeal PurF candidates require cleavage of several residues to reveal the N-terminal cysteine and others require only removal of the N-terminal methionine. Although experimental data is required to verify these translation initiation sites, our findings are consistent with at least 5% error rate (3/58) for initiation site determination in these *purF *genes.

Alignment and phylogenetic tree generation for the two PurF-like proteins in each of the Crenarchaeota revealed that two distinct clusters (Additional Files 1 and 2), with each Crenarchaeon having one gene in each cluster. One cluster contained proteins with the expected active-site N-terminal cysteine, and we believe these are the actual PurF enzymes. The proteins in the second cluster, which we call PurF', lacked the N-terminal cysteine necessary for glutaminase activity. Many PurF' proteins had alanine or valine, which could not substitute as the nucleophile required for glutaminase activity. These *purF' *gene loci are enclosed in parentheses in Additional File 1 and denoted with "□" in Figures 2 and 3 to indicate the missing active site feature. The genes *purF *and *purF' *are close to each other and to other purine biosynthesis genes in these genomes; thus, gene clustering supports the role of both PurF and PurF' in purine biosynthesis. Many of the Crenarchaeota have other ORFs that are bidirectional best hits for the similar amidotransferases AsnB and GlmS, so we do not believe that either of these *purF*-like genes is actually *asnB *or *glmS *[23]. As both PurF and AsnB exhibit a loss of function when the equivalent cysteine residue is replaced with alanine or serine [24], the PurF' proteins are unlikely to be functional amidotransferases with any substrate. One possibility is that PurF' acts an ammonia-dependent phosphoribosylpyrophosphate aminotransferase, a hypothesis that will require experimental testing.

We identified an additional C-terminal domain in *Methanospirillum hungatei's *PurF. This C-terminal domain is a HEPN-type domain, which might be involved in nucleotide binding. This feature was unique to *M. hungatei*, and its specific function in this PurF candidate is unclear.

### Glycinamide ribonucleotide synthetase (EC 6.3.4.13, GARS, PurD)

Glycinamide ribonucleotide synthetase, PurD, is an ATP-grasp protein, responsible for conversion of 5-phospho-β-D-ribosylamine (PRA) to 5-phosphoribosylglycinamide (GAR), with incorporation of glycine and hydrolysis of ATP to ADP. Transfer of the unstable PurD substrate, PRA, from PurF is believed to occur through a transient interaction between these two enzymes [25]. Studies of PurF and PurD from *Aquifex aolicus *indicated that coupling occurs, but the measured *in vitro *efficiency was too low to be biologically useful, suggesting that another protein or small molecule may be required to reconstitute a functional assembly [26].

A gene encoding a protein similar to PurD was identified in all purine-synthesizing archaea. Two candidates for PurD were present in *Haloarcula marismortui *(rrnAC1109 and rrnAC0307) and *Halorubrum lacusprofundi *(Hlac_1295 and Hlac_1553). We suspect rrnAC1109 and Hlac_1295 encode the functional PurD enzymes, based on problems with Hlac_1553 and rrnAC0307, including missing Pfam GARS_N sequences and Mg^2+ ^binding sites. These two "extra" PurD-like proteins were the only PurD-like sequences we identified in which a highly conserved aspartate in the R[LF]GDPEx[EQIM] motif (Prosite PS00184, corresponding to amino acids 290-298 in the *E*. coli PurD) was not conserved [27]. Similarly, the N-terminal P-loop region is quite divergent in Hlac_1553 and rrnAC0307 compared to the other archaeal PurD-like proteins. We conclude that these "extra" proteins are not actual glycinamide ribonucleotide synthetases, despite their annotations.

### Formate-dependent GAR formyltransferase (EC 6.3.4.-, PurT) and GAR formyltransferase (EC 2.1.2.2, PurN)

Conversion of GAR to formyl glycinamide ribonucleotide (FGAR) may be catalyzed by either of two distinct formyltransferases. The first, PurN, transfers a formyl group from 10-formyltetrahydrofolate to GAR. The second, PurT, uses formate as the one-carbon source for formylation of GAR, with coupled hydrolysis of ATP. In those archaea that use only methanopterin-related coenzymes as C-1 carriers, with no 10-formyltetrahydrofolate present [28], a *purN *gene is not expected, as PurN requires 10-formyltetrahydrofolate as the one-carbon donor. Although superficially similar to 10-formyltetrahydrofolate, N^10^-formyltetrahydromethanopterin is not believed to be functional as a formyl group donor [29].

GAR formyltransferase activity appears to be provided by PurN in the Thaumarchaeota, Thermoplasmata, Methanomicrobiales, Methanosarcinales, Thermoproteales, and Halobacteriales. The residues involved in catalysis in the *E. coli *enzyme (His108 and Asp144 [30]) are conserved in each of these archaeal enzymes. *Methanosarcina barkeri *has been previously determined to contain tetrahydrofolates [31], which would be required for PurN activity. Tetrahydrofolates are expected to be present in all PurN-containing organisms. The incorporation of labeled acetate at C8 of purine synthesized in *M. hungatei *and *M. barkeri *is consistent with this formyl group being transferred from 10-formyltetrahydrofolate by a PurN-type GAR formyltransferase [32], consistent with the presence of *purN *in the genomes of these organisms.

The nine species of Halobacteria studied have a PurN-like enzyme fused to a PurH1-like domain (which would also require tetrahydrofolates). Some of these organisms have previously been shown experimentally to contain tetrahydrofolates [33]. *H. walsbyi *has both the *purN-purH1 *fusion and a *purT*-like gene. It is unknown whether both genes encode functional enzymes, providing *H. walsbyi *with two alternatives depending on growth conditions (as occurs in *E. coli*), or whether one gene is non-functional. The *purT *gene seems likely to be a recent acquisition by horizontal gene transfer, as *H. walsbyi*'s closest relatives have only PurN for GAR formyltransferase activity, and the *H. walsbyi *PurT sequence is more closely related to bacterial PurTs than to other archaeal PurT sequences. In our consensus phylogenetic tree for PurT (Additional File 2), the *H. walsbyi *protein was a deep branch near the center of the unrooted tree.

The PurT enzyme (previously identified in bacteria [4]) provides GAR formyltransferase activity in archaea that have no tetrahydrofolates, including as *Methanocaldococcus jannaschii, Methanococcus maripaludis*, and *Pyrococcus *spp. A *purT*-like gene is also present in the Sulfolobales, which are believed to have very low levels of tetrahydrofolates [33] along with a folate analogue with characteristics of both tetrahydrofolate and methanopterin [34]. The active site residues of the putative archaeal PurT enzymes are identical to those identified in *E. coli*, with complete conservation at positions 114, 155, 160, 162, 195, 203, 267, 279, 286, 355, 362, and 363 (*E. coli *numbering) [35].

In *E. coli*, the function of PurT for purine biosynthesis requires the presence of formyltetrahydrofolate hydrolase, PurU, to provide PurT with formate from 10-formyltetrahydrofolate. Thus, *E. coli *deletion mutants *purN purT *and *purN purU *were unable to convert GAR to FGAR, while single gene deletions did not block production of FGAR [36]. In *E. coli*, both GAR formyltransferase variants -- the PurN-catalyzed reaction using 10-formyltetrahydrofolate and the PurT-catalyzed reaction using formate from PurU -- require 10-formyltetrahydrofolate. In those archaea with only archaeal folate analogues (such as methanopterin), purine biosynthesis must proceed differently. Although a PurT enzyme may be functional in these organisms, its formate cannot be supplied by PurU.

A PurU homologue is absent from nearly all of the PurT-utilizing archaea. These archaea apparently have enough formate present not to require channeling from a PurU enzyme. We did identify a PurU-like protein in each of the Halobacteria and Thaumarchaeota, with conserved catalytic residues His 106 and Asp 142 (human numbering) [37]. The presence of PurU in these species, given that only *Haloquadratum walsbyi *has a candidate for PurT, was surprising. This PurU-like protein may regulate levels of folates, as proposed for *E. coli *[38], or may produce formate for another purpose in these archaea. A purine-related role is suggested by the genomic location of the *purU*-like gene in some of the Halobacteria, where it was found in a gene cluster with *purS, purQ*, and *purC*. The two Thaumarchaeota may be using PurU to produce formate for PurP (discussed below), but the Halobacteria do not contain PurP, and thus there is no clear reason for a formate-producing enzyme's gene to cluster with purine biosynthesis genes in the Halobacteria.

We could not identify a GAR formyltransferase (*purN*/*purT*) gene in seven species of Archaea that appear to have otherwise intact purine biosynthesis pathways. The two Archaeoglobi, four Methanomicrobiales, and *Methanopyrus kandleri *have no genes with high similarity to known *purT *or *purN *genes despite otherwise complete or near-complete purine biosynthesis pathways. *Archaeoglobus fulgidus *and *Methanothermobacter thermoautotrophicus *are known to contain methanopterin-related folate analogues rather than tetrahydrofolates, so the unidentified enzymes might use formate as a carbon source (as in PurT), or may use an alternate non-folate carbon source other than methanopterin, as discussed above. Although *Methanosphaera stadtmanae, Methanobrevibacter smithii*, and *Methanobrevibacter ruminantium *are intestinal commensals and thus might rely on an environmental source of purines (despite the presence of an otherwise complete pathway), *M. thermoautotrophicus *is an established purine prototroph [39] and so must have some means to catalyze the GAR formyltransferase reaction. Labeling studies are inconsistent as to the substrate of the missing GAR formyltransferase in the Methanomicrobiales. In the presence of formate, *M. stadtmanae *has been shown to derive C8 from C2 of acetate (most consistent with the use of a tetrahydrofolate C1 carrier), while closely related *M. smithii *does not derive C8 from acetate [32].

### Phosphoribosylformylglycinamidine synthetase (EC 6.3.5.3, PurL, PurS, PurQ)

Phosphoribosylformylglycinamidine synthetase (FGAM synthetase or FGAR aminotransferase) catalyzes the synthesis of FGAM from FGAR, glutamine, and ATP. FGAM synthetase has been observed to occur as either a single multidomain enzyme (lgPurL) or as a multiprotein complex composed of a shorter PurL (smPurL) corresponding to the central domain of lgPurL and two other proteins: PurQ, a glutaminase similar to the C-terminal glutaminase domain in lgPurL, and PurS, which is structurally similar to the N-terminal domain of lgPurL [40-42]. The PurS protein is required for purine biosynthesis in *B. subtilis *[5] and presumably in other organisms with smPurL. PurS has been proposed to be a "protein-protein interaction module" [43], but there is disagreement in the literature about its physiologically relevant form [40]. Most of the purine-biosynthesizing archaea we studied had genes encoding smPurL, PurQ, and PurS, similar to the situation in Gram-positive bacteria.

In *M. labreanum*, a large gene encoding a protein similar to lgPurL, Mlab_0300, is present. This gene is more closely related to the *lgpurL *genes in Clostridia and other bacterial species than it is to any gene in the sequenced archaea. Separate genes for *purS *and *purQ *are not present in *M. labreanum*, as would be expected for an organism with a lgPurL. This result is consistent with the gene being horizontally transferred from *Clostridia *after divergence of *M. labreanum *from its nearest sequenced relatives. We found Mlab_0300 alternately annoted as "carbamoyl-phosphate synthetase, large subunit" or FGAM synthase in the databases consulted. We did not see compelling evidence for a carbamoyl phosphate synthetase assignment for this gene.

*Methanospirillum hungatei, Methanoculleus marisnigri, Methanocella sp*. RC-I, *Methanoregula boonei, Methanococcus vannielii*, and *Methanococcus maripaludis *encode a form of PurL that is longer than the other smPurLs seen in most archaeal species. The larger gene product (which we call mdPurL) has approximately 200 additional amino acids at the N-terminus, where a PurS-like domain would occur in lgPurL. In these species, the N-terminal sequence shares only low sequence identity with the similar portion of lgPurL or PurS, but we suspect that this domain serves a similar structural function to PurS or the N-terminal portion of lgPurL. Five of the six organisms with mdPurL lack a *purS *gene, supporting this supposition. *M. marisnigri *has both *mdpurL *and a *purS *gene candidate, in what may be an example of redundancy. The pattern of distribution of *mdpurL *versus *smpurL *plus *purS *is patchy, with closely related species having different *purL *lengths.

The catalytic triad [41] of the glutaminase (PurQ, or the corresponding domain of lgPurL) is present, with the cysteine and glutamate fully conserved, and conservation of histidine in all sequences except the two Thaumarchaeota, where an asparagine is present. We also identified an additional PurQ-like protein in *M. marisnigri*, Memar_1841. This putative protein lacks roughly 150 residues, including the all three residues in the catalytic triad. Memar_1841 is clustered with the candidate genes for *purS *and *purC*, while the full-length *purQ*, Mmar_1628, is adjacent to the gene encoding the mdPurL candidate. We suspect a duplication event or horizontal gene transfer (HGT) followed by gene decay, which would serve to explain both the extra fractional *purQ *and an unexpected *purS *gene.

PurS proteins are typically less than 100 amino acids in length. We had problems with earlier versions of several databases with finding PurS-like ORFs, which necessitated searching for missed ORFs with TBLASTN (data not shown). The problem of unidentified ORFs appears to be resolved for PurS, but serves to illustrate the danger of using arbitrary minimum lengths as a cutoff in coding sequence identification. We may be missing additional small but important proteins.

### Phosphoribosylformylglycinamidine cyclo-ligase (AIR synthetase, 6.3.3.1, PurM)

PurM, aminoimidazole ribonucleotide (AIR) synthetase, catalyzes the conversion of FGAM to AIR, with hydrolysis of ATP to ADP. PurM is structurally and mechanistically related to the central domain of PurL (smPurL), the enzyme in the previous step of purine biosynthesis [44,45]. We identified one candidate gene for *purM *in each of the purine-biosynthesizing organisms studied, with bidirectional best hits on *E. coli *PurM. Three previously proposed [46] active site residues, His190, Asp65, and Asp94 (*E. coli *numbering), are 100% conserved in the species studied, while replacement of His 247 with asparagine occurs in *Caldivirga maquilingensis, Thermoplasma acidophilum, Picrophilus torridus*, and *Thermoplasma volcanium*. As there is no other obvious candidate for PurM in these species, we suspect that the H247N change does not significantly impair the ability of this enzyme to catalyze the reaction.

### Conversion of AIR to CAIR (AIR carboxylase, 4.1.1.21, class II PurE; NCAIR synthetase, 6.3.4.18, PurK; and NCAIR mutase, 5.4.99.18, class I PurE)

In eukaryotes, a class II PurE enzyme effects formation of 5-amino-4-imidazole-carboxylic acid ribonucleotide (CAIR) directly from AIR by addition of CO_2 _[47], with no ATP requirement. However, the conversion of AIR to CAIR in bacteria requires two enzymes, PurK and class I PurE. The PurK enzyme uses AIR, bicarbonate, and ATP to produce the unstable 5-carboxyamino-1-(5-phospho-D-ribosyl)imidazole (*N*^5^-CAIR) intermediate and ADP. The *N*^5^-CAIR is then converted to CAIR by a class I PurE enzyme. Although *N*^5^-CAIR is unstable, PurE and PurK have not been shown to associate *in vitro *[6].

While a *purE *gene candidate is present in all purine biosynthesizing archaea studied, the distribution of *purK *is patchy, as shown in Tables 1, 2, with *purK *present in most Crenarchaeota, in the Halobacteria, in some Thermococci, and in the Thermoplasmatales. The absence of *purK *from the methanogens, *Ignicoccus hospitalis*, and *D. kamchatkensis *is consistent with high availability of CO_2 _in these species' environments. For those organisms that do contain a PurK enzyme, we found the active site residues identified by Thoden and coworkers [48] to be well conserved. Lys 120 (*E. coli *numbering), which contacts the adenine ring of ATP, is 100% conserved in archaeal PurK. Glu 153, another adenine-contacting position, is fully conserved except for two cases where it is substituted with glutamine and one case with aspartate, both fairly conservative substitutions. Arg 80, which contacts the ATP phosphates, is present in the Crenarchaeota, but conservatively substituted with lysine in Thaumarchaeota and PurK-containing Euryarchaeota. Gln 11, proposed to contact the AIR substrate, is fully conserved after correcting for an incorrect translation initiation site in *Halobacterium sp*. NRC-1. An insertion in the Crenarchaeota and generally low homology in the region of Glu 49, thought to make contacts to the ribose ring of AIR, prevented us from making a definitive determination about conservation of this residue. Lys 307, Arg 313, and Lys 314, postulated to contact AIR, and Asp 127, postulated to act as a base during the reaction, were fully conserved.

**Table 1:** Annotation errors identified, by gene

	*pur *genes																
	F	D	N	T	L^a^	S^a^	Q^a^	M	K	E	C	B	H1^c^	P	**P2**^d^	H2	O
**Protein name errors**																	
Partial misannotation/over-attribution			3			2			1	^b^							
Inappropriately vague name						23				1	1			27		1	4
Not justified due to missing features	14	2					1								4		
**E. C. number errors**																	
One or more missing			6			13			23	35	1						
One or more incorrect/unjustified	14	2	2	2						2			1	1	31	2	
**Gene structure errors**																	
Start codon mis-called	3								1								
Pseudogene label unjustified											1						
**Gene symbol errors**																	
Incorrect gene symbol							2				1					1	1
**Number of genes examined**	72	60	31	23	58	51	58	58	28	59	63^e^	60	13^c ^	46^e^	31	2^f^	25

^a ^The three gene products share an EC number.

^b ^Naming of PurE is problematic. Some PurEs are not clearly class I or class II, and some organisms lack a PurK, making a class II type name more appropriate even when PurE appears class I. We counted either a class I or class II-type name as correct in this analysis.

^c ^Halobacteria fusions of PurN and PurH1 are counted under PurN.

^d ^Separate counts were maintained for PurP-like proteins in cluster II. We preferred generic names and no EC number, given a lack of demonstrated function for proteins in this cluster.

^e ^A split or frame-shifted gene was counted as one gene.

^f ^Excludes full-length PurH, counted under PurH1.

There may be an evolutionary advantage for the use of class II PurE or non-enzymatic production of *N*^5^-CAIR, with avoidance of ATP hydrolysis by PurK, but only in the context of a sufficiently high concentration of CO_2 _to give a reasonable rate of CAIR synthesis. *E. coli *PurE has been shown to catalyze conversion of AIR to CAIR (without PurK) in the presence of high concentrations of bicarbonate [6]. It has been suggested that PurK may not be required for hyperthermophilic organisms growing at high CO_2 _concentrations, where the non-enzymatic conversion of AIR to *N*^5^-CAIR may be sufficient [49]. Patrick *et al*. have demonstrated complementation of the Keio collection *E. coli purK *deletion with over-expression of plasmid-encoded PurE, which they suggest helps shift the equilibrium position of the non-enzymatic reaction, compensating for lack of PurK [50]. We thus do not consider the absence of *purK *to be an indication that an unknown gene is involved in the pathway, so much as a reflection of a variety of strategies for production of CAIR depending on carbon dioxide availability.

*Archaeoglobus profundus *and *A. fulgidus *have a class II-like PurE and no PurK. These PurEs from the Archaeoglobi have the six conserved distinguishing residues for class II enzymes as proposed by Matthews *et al*. [51], as previously described for the *A. fulgidus *protein. The *purE *genes appear to have been horizontally transferred from a eukaryote, with loss of the ancestral organism's class I *purE*, as the present genes encode proteins more closely related to eukaryotic PurEs than to any other archaeal PurEs.

The four Methanobacteria (*M. smithii, M. thermoautotrophicus, M. ruminantium*, and *M. stadtmanae) *have a gene encoding two fused PurE-like domains, but no *purK *gene. The *M. thermoautotrophicus *and *M. smithii purE-purE' *genes complement both *purE *and *purK *deletions in *E. coli *[52], suggesting that a *purK *gene is not necessary in *M. thermoautotrophicus *or *M. smithii*. The first domain (PurE) has been characterized as class I based on amino acid sequence [51]. Intriguingly, a deletion in the second half of *M. smithii *gene (the *purE' *region) removed the ability of that gene to complement either *purE *or *purK *deletions in *E. coli *[52], suggesting that it may actually be the PurE' that is involved in catalysis of both steps of this reaction, despite lower similarity of this second domain to known PurE enzymes. Alternately, the PurE' domain might be structural in nature but strictly required for subunit assembly. Regardless of which domain is responsible for the catalysis, we conclude that all four of these double-length PurE-like proteins effect the AIR carboxylase reaction alone on the basis of the complementation results with the *M. smithii *and *M. thermoautotrophicus *genes.

The other methanogens, the Desulfurococcales, and some of the Thermococci have a class I-like PurE and no PurK. With the exception of a methionine at position 14 (*E. coli *numbering), which is not conserved in archaea, the other conserved positions in our PurE candidates match those identified by Mathews and coworkers [51]. These class I-like PurE enzymes are either using high concentrations of carbon dioxide and AIR as substrates, are using *N*^5^-CAIR produced from non-enzymatic reactions, or have an alternate enzyme involved in *N*^5^-CAIR production.

The Halobacteria studied have a PurE-like protein that we could not unambiguously assign to either class I or class II. An insertion after the first helix (relative to the *E. coli *structure), a lysine at position 73, and poor alignment in the otherwise conserved region at the end of helix 4 result in no match to the consensus sequence of class I or class II. As these organisms have a separate *purK*-like gene, these PurE enzymes only need to perform the class I function, which we expect based on these organisms' occurrence in cool, aerobic environments.

Except for the Desulfurococcales, the Crenarchaeota have a class I PurE accompanied by PurK. Experimental support for this assignment is available in *Sulfolobus solfataricus*, in which the *purE *and *purK *genes have each been previously shown to complement *E. coli *with the corresponding deletion, with no ability of the *S. solfataricus purE *gene to complement *E. coli purK *deletions or vice versa [53]. High temperature alone thus does not appear sufficient to remove the utility of *purK *in the absence of high carbon dioxide levels.

### Phosphoribosylaminoimidazole-succinocarboxamide synthetase (EC 6.3.2.6, SAICAR synthetase, PurC)

The *purC *gene product, SAICAR synthetase, catalyzes the conversion of L-aspartate, ATP, and CAIR into phosphoribosylaminoimidazole-succinocarboxamide (SAICAR), ADP, and phosphate. PurC is structurally and mechanistically related to adenylsuccinate synthase (PurA), which converts IMP, L-aspartate and GTP to adenylsuccinate, GDP, and phosphate [54]. *E. coli *PurC has been kinetically characterized [54], and structures of PurCs from several species have been reported [55-57] or deposited (MJ1592).

We identified at least one *purC*-like gene in all purine-synthesizing archaeal species. Conservation of the substrate-contacting residues identified by Ginder and coworkers [57] for the *E. coli *crystal structure with bound CAIR and ADP is high. Glu 179, which contacts ADP, and Ser 100, Arg 94, Arg 199, and Asp 175, which contact CAIR, are fully conserved. However, Lys 11 and Lys 13, which make contacts to CAIR, are not present in the Halobacteria or in the Archaeoglobi. Gln 69 is not conserved in the archaea, but a highly conserved histidine is present at the corresponding position in the archaeal proteins. CAIR-contacting residue Arg 215 is conserved or conservatively replaced with Lys, except in *Thermococcus gammatolerans *(TGAM_1268), *Thermococcus kodakaraensis *(TK0432), and *M. labreanum *(Mlab_0676), where the predicted coding sequence ends before this position. Interestingly, the two Thermococci have two *purC*-like genes, so it is possible that the truncated *purC*-like gene does not encode active enzyme. *M. labreanum*'s Mlab_0676, however, is the organism's only apparent *purC*, suggesting that SAICAR synthetase activity may be possible even when PurC is truncated.

We built a maximum likelihood phylogenetic tree for the predicted PurC-like proteins, including a selection of eukaryal and bacterial sequences, based only on well-aligned regions (Additional File 2). Bootstrap values were low, thus interpretation of the results merits caution. Neither the Crenarchaeota nor Euryarchaeota were monophyletic in our analysis, nor were the selected eukaryal and bacterial sequences. Within the limitations of the trees built, the history of *purC *does not seem to match the history of the organisms examined. This could be caused by numerous horizontal transfers or by distinctly different selective pressures on these genes, such as if some proteins are not actually SAICAR synthetases or have a second activity in addition to SAICAR synthetase. At least two closely related reactions, conversion of IMP to adenylosuccinate (catalyzed by PurA) and synthesis of β-RFSA-P (4-(β-D-ribofuranosyl)-N-succinylaminobenzene 5'-phosphate) from phosphorylated β-RFH-P (4-(β-D-ribofuranosyl)hydroxybenzene 5'-phosphate) [58] require very similar chemistry and may be the proposed additional or alternate functions for some of the proposed PurC proteins.

The predicted PurC protein in *M. hungatei *(Mhun_1253) is unusual, in that it includes an N-terminal radical SAM (S-adenosylmethionine) binding domain similar to CofG [59] (49% identical to *M. jannaschii *CofG, 60% identical to a suspected FO synthetase subunit I in *M. labreanum*), in addition to a C-terminal PurC-like domain. While we did not find this fusion in other archaeal species, the *purC*-like genes in *Methanosphaerula palustris, M. boonei*, M. *labreanum, M. barkeri *(Mbar_A3291), *M. marisnigri, T. kodakaraensis *(TK0432), and *T. gammatolerans *(TGAM_1268), each have a gene with a radical SAM-like motif immediately preceding them on the same strand. We also found a nearby gene on the opposite strand with a radical SAM-like motif (sometimes annotated as *cofH*) in several of the Halobacteria. Although the involvement of S-adenosylmethionine in purine biosynthesis has not been proposed, S-adenosylmethionine is required for thiamine biosynthesis, which uses the purine biosynthetic intermediate AIR. S-adenosylmethione is also required for the conversion of GTP to the FO cofactor. Thus, this radical SAM-binding motif might represent a regulatory domain for the PurC enzyme, or might code for an enzyme required for one of these other biosynthetic processes but co-regulated with PurC due to the need for purines for cofactor biosynthesis.

We observed apparent duplications of the *purC *gene in *Methanococcus aeolicus, M. barkeri, T. gammatolerans*, and *T. kodakaraensis*. Neither of the *M. aeolicus *genes is close to other purine biosynthesis gene candidates in the genome. Based on 74% sequence identity and well-conserved (82% BV) phylogenetic grouping of the two *M. aeolicus *proteins, we concluded that this is a relatively recent duplication. In *M. barkeri*, one *purC *candidate, which is split into two adjacent loci (Mbar_A0530 and Mbar_A0531) is near the *purL *gene, while the second (Mbar_A3291) is not clustered with other purine biosynthesis genes and is ambiguously annotated as "Phosphoribosylaminoimidazole carboxylase, phosphoribosylaminoribosylaminoimidazole succinocarboxamide synthetase." This second *M. barkeri *protein and the only PurC protein from *Methanocella sp. RC-I *are sister proteins on the phylogenetic tree, with closer relationships to the PurC proteins in eukaryotes than to other archaeal PurC proteins. We did not find any evidence that *M. barkeri *gene encodes a protein with PurE character, so we concluded that this is a misannotation probably resulting from incorrect transfer of the annotation of a fused PurCE protein (common in eukaryotes). In *T. kodakaraensis *and *T. gammatolerans*, one gene candidate for *purC *is near *purM, purT*, and *purF*, while the other, which shows some evidence for HGT based on our phylogenetic tree, is near *purO *and one of the *purP *candidates, suggesting that both *purC-*like genes are associated with purine biosynthesis.

### Adenylosuccinate lyase (EC 4.3.2.2, PurB)

Adenylosuccinate lyase (PurB) plays a dual role in purine biosynthesis. This enzyme catalyzes two different β-eliminations, accomplishing conversion of SAICAR to AICAR and of adenylosuccinate to AMP. Both reactions produce fumarate. Structural studies reveal that a single active site catalyzes both reactions [60], consistent with the very similar chemistry involved. Given the participation of PurB in purine interconversion, we expect to find a *purB *gene in some organisms that lack the *de novo *biosynthesis pathway but are capable of purine interconversion.

We identified one gene candidate for *purB *in each of the purine biosynthesizing archaea. Among the active site residues previously identified [60], His 143 is completely conserved among the archaeal species studied, as are Glu 275, Asn 270, Gln 216, and His 72. *M. jannaschii *PurB was recently confirmed to have the ability to convert adenylosuccinate to AMP in addition to catalyzing the conversion of 4-(β-D-ribofuranosyl)-N-succinyl-aminobenzene 5'-phosphate (β-RFSA-P) to 4-(β-D-ribofuranosyl)aminobenzene 5'-phosphate (β-RFA-P) in the biosynthesis of the arylamine of methanopterin [58]. It is not yet known whether all PurBs from methanopterin-utilizing organisms also participate in the synthesis of β-RFA-P.

A phylogenetic tree of well-conserved regions of the PurB sequences contained mostly the expected organization at the order level and below, although bootstrap values near the putative root were low. With one exception, the Crenarchaeota were monophyletic. Our consensus tree had all of the halobacterial PurB candidates and the PurB candidate from *Caldivirga maquilingensis *in a cluster with eukaryal and bacterial PurBs, with the closest relationship being with *Chloroflexus*. The tree structure could have been caused by two horizontal gene transfers, an ancient one in the last halobacterial ancestor, and a more recent one from a halobacterium to *Caldivirga maquiligensis*. The observed tree structure could also be caused by the incorrect assignment of all of these proteins to the PurB function. Characterization of archaeal PurBs has been limited to the *Methanocaldococcus jannaschii *enzyme, which as described above, has had an additional "moonlighting" activity demonstrated in addition to one of the two canonical PurB activities. Some of the unusual relationships in our phylogenetic tree may be caused by differences in substrate specificity.

The *purB *gene is clustered with the fused *purH1N *gene in the Halobacteria and with the two *purP *candidate genes (discussed below) in the Sulfolobales and *Caldivirga maquilingensis*. In both cases, the *purB *gene is in the opposite orientation from the *purP *or *purH1N *gene, suggesting bidirectional transcription from a shared promoter site between the two genes. The similar gene arrangement is surprising, given that the Sulfolobales and Halobacteria are not closely related and that the AICAR formyltransferases PurP and PurH1 are structurally and mechanistically unrelated despite effecting the same biosynthetic step.

In their crystal structure of the *Pyrobaculum aerophilum *PurB, Toth and coworkers identified three intrachain disulfide bonds that stabilize the enzyme [60]. These disulfide bonds are present only in the genus *Pyrobaculum *and are not a general feature of archaeal or thermophile PurB. Although the methanogens and Thermococci have cysteine-rich PurBs, these cysteines are not aligned with those of *P. aerophilum *PurB. The *M. jannaschii *PurB has eight cysteines, only some of which appear to form one or more disulfides, as a result of air oxidation during purification. This oxidation greatly reduces enzymatic activity. (White, unpublished results). The reason for the enzyme having so many cysteines remains to be elucidated.

### Aminoimidazole carboxamide ribonucleotide formyltransferase (folate-dependent AICAR formyltransferase, EC 2.1.2.3, PurH1 or formate-dependent AICAR formyltransferase, EC 6.3.4.-, PurP) and IMP cyclohydrolase (EC 3.5.4.10, PurH2/PurO)

In eukaryotes and bacteria, aminoimidazole carboxamide ribonucleotide (AICAR) formyltransferase and IMP cyclohydrolase activities occur as the bifunctional enzyme PurH. Deletion studies on the avian gene revealed that PurH has two separate catalytic sites and that each half of the enzyme catalyzes one step in isolation [61]. Except in the archaea, the two domains commonly occur fused as a single bifunctional enzyme, alternately called PurH or PurHJ (with "PurJ" representing the N-terminal IMP cyclohydrolase domain) [46]. To avoid confusion over varying usage of "PurH", in this manuscript we have used the term "PurH1" for the C-terminal AICAR formyltransferase (which occurs first in the pathway) and "PurH2" for the N-terminal IMP cyclohydrolase.

The PurH1 AICAR formyltransferase domain uses 10-formyltetrahydrofolate to transfer a formyl group to AICAR, producing FAICAR. The IMP cyclohydrolase (PurH2) domain of PurH catalyzes the cyclization of FAICAR to IMP, with loss of water. Given the requirement for 10-formyltetrahydrofolate (and not an archaeal folate analogue), a different enzyme must participate in the conversion of AICAR to FAICAR in non-folate-containing archaea. The search for AICAR formyltransferase activity in these archaea led to the discovery that the archaeal signature *purP *gene product served this function in some archaeal species, using formate as a carbon source [8].

The PurO enzyme, from the archaeal signature *purO *gene, has been shown to catalyze the IMP cyclohydrolase reaction in *M. jannaschii *and presumably also in related organisms [7]. Although the catalytic mechanism of PurO appears to be similar to that of PurH2, the protein structures and sequences are unrelated [62]. Together, PurP and PurO can act as a replacement for full-length PurH in *M. jannaschii*, requiring ATP and formate in place of 10-formyltetrahydrofolate.

There is a great deal of biosynthetic diversity among the archaea in the final two steps of the *de novo *pathway. We found all possible combinations of the AICAR formyltransferases PurH1 and PurP with the IMP cyclohydrolases PurH2 or PurO, along with instances of both apparent redundancy in the AICAR formyltransferase step and missing genes for the IMP cyclohydrolase step.

The Methanomicrobiales studied have a full length *purH*-like gene (encoding both PurH1 and PurH2 domains) and no genomic evidence for *purP *or *purO*. Except for *Methanosaeta thermophila*, the Methanosarcinales also have a full-length *purH*-like gene, in addition to multiple genes (typically two) encoding a protein similar to the well-characterized *M. jannaschii *PurP [8]. It is unclear whether these PurP-like proteins are functional in these species, or whether the PurH-like protein catalyzes the necessary conversions alone. Given the presence of a *purN *gene and experimental determination that *M. barkeri *uses both folates and archaeal folate analogs [31], all evidence indicates that use of 10-formyltetrahydrofolate as a source of formyl units is possible in these organisms, consistent with a functional PurH1 domain. Label incorporations studies in *M. barkeri *and *M. hungatei *are consistent with catalysis of the formyltransferase reaction by PurH1, with the formyl group derived from a tetrahydrofolate carrier [32], although the low levels of label incorporation in *M. barkeri *do not preclude activity of a PurP enzyme as well.

Active site residues in both PurH1 and PurH2 were typically conserved in the archaea that contain these domains. The residues identified as being important to the IMP cyclohydrolase domain of human PurH (Lys 66, Tyr 104, Asp 125, and Lys 137 [63]) are fully conserved in the fourteen archaea containing a PurH2 domain. In the PurH1 domain, the residues contacting AICAR (Arg 208, Tyr 209, Lys 267, His 268, Asn 342, Arg 352, Phe 342, and Arg 589, human numbers [64]) are fully conserved, while Asp 340 has been replaced by Glu in all archaea containing a PurH1 domain.

*M. thermophila*'s PurH-like protein (Mthe_1511) does not have the expected N-terminal PurH2 domain, but instead has 130 residues N-terminal to the PurH1 domain that have no evident homology to an ORF in any other sequenced genome. A region of gene duplication occurs immediately upstream of the Mthe_1511 ORF (locus tags Mthe_1512-1532). The event that caused this duplication may also have removed *purH2*. A *purO *gene, which could serve as a replacement for the missing *purH2 *is present in *M. thermophila *but not in closely related species. The remaining (PurH1-like) protein encoded by Mthe_1511, however, is not particularly similar to the PurH1 domain from other Methanosarcinales, being more similar to several bacterial species, which may indicate a more complex gene heritage than a simple domain loss.

Although the avian PurH1 and PurH2 domains exhibit activity when expressed separately, only in the Archaea do we find isolated PurH domains frequently occurring. In the Halobacteria, only the PurH1 domain is present, as a fusion to PurN. The presence of PurH1 is consistent with the experimentally established existence of tetrahydrofolates in some of these organisms [33]. In these species, a *purO*-like gene is present, whose gene product we expect to catalyze the necessary IMP cyclohydrolase reaction based on similarity to the *M. jannaschii *PurO enzyme. In the Archaeoglobi, the situation is reversed. Archaeal PurP-like proteins are present, along with a gene encoding a PurH2-like protein. Here, PurH2 performs the IMP cyclohydrolase activity (Hunter, Plymale, and Sarisky, unpublished results), with a PurP protein suspected to provide folate-independent formyltransferase activity. A PurH1-domain could not be functional in *A. fulgidus*, as non-archaeal tetrahydrofolates are not present [65].

**Figure 5:**
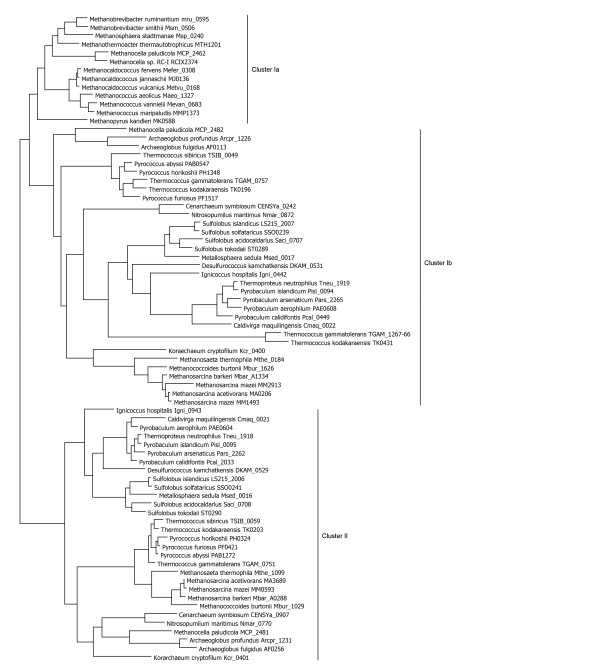
**Maximum likelihood phylogenetic tree for PurP-like proteins**. The species name is followed by the locus tag. For clarity, bootstrap values are not shown, but are available in Additional File 2.

Many archaeal genomes contain two *purP*-like genes. Phylogenetic trees constructed using all studied PurP-like archaeal proteins revealed clusters of protein sequences, shown in Figure 5. Cluster Ia was monophyletic in all of our analyses, and included the experimentally characterized *M. jannaschii *PurP enzyme, the single PurP-like protein from each of the Methanococcales, Methanobacteriales, and *Methanocella sp*. RC-I, and one of the PurP-like proteins (MCP_2462) from *Methanocella paludicola*. As each of these organisms (except for *M. paludicola*) has a single PurP with high similarity to *M. jannaschii *PurP and no PurH1, all are presumed to have the expected formate-dependent AICAR formyltransferase (PurP) activity. Cluster Ib contains a more divergent group of proteins. In some analyses, cluster Ib was monophyletic, but in others the consensus tree placed cluster Ia as a subtree of the cluster Ib region. Cluster Ib contains predicted PurP-like proteins from the purine-biosynthesizing Thermococci, Crenarchaeota, Thaumarchaeota, Archaeoglobi, and Methanosarcinales. Most of these organisms have one PurP-like protein in this cluster. *Methanosarcina mazei *has two closely related proteins in cluster Ib, while *T. kodakaraensis *and *T. gammatolerans *each have one protein closely related to the cluster Ib proteins of other Thermococci and one protein (TK0431 and the split TK1267 and TK1266) that is only distantly related to other PurP proteins in cluster I. The "extra" *T. kodakaraensis *gene product has been crystallized without substrates (PDB code:2pbz) but awaits enzymatic characterization. Cluster II contains exactly one protein each from the species with a representative in cluster Ib. While the cluster Ia/b and cluster II subtrees each are similar to accepted phylogenetic trees, the combined consensus tree is consistent with gene duplication and divergence of these *purP-*like genes pre-dating the divergence of these archaea from a common ancestor. Thus, we propose that both cluster Ia/b and cluster II-type *purP *genes were present in the last archaeal common ancestor, with patchy loss of one or both *purP*-like genes in some of the Euryarchaeota.

In inspecting the sequence alignment for the PurP-like proteins in both clusters, we found 100% conservation of Arg 314 and His 27 (*M. jannaschii *numbering), which make contacts to the formyl group in the MJ0136 crystal structure [66]. Residues contacting the 5' monophosphate of AICAR were largely conserved, although we found conservative substitution with Thr at Ser 94. Ser 94 was not conserved in Kcr_0401, while Ser 266 was not conserved in Kcr_400. Asn 258, which contacts the carboxamide of AICAR, was completely conserved in cluster Ia/b and but was always histidine in the cluster II.

**Figure 6:**
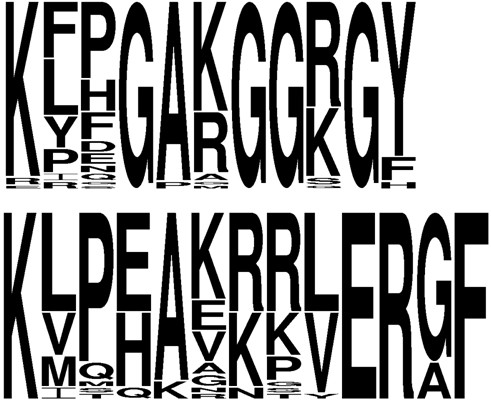
**WebLOGOs for the combined cluster Ia/Ib P-loop (top) and corresponding cluster II loop region (bottom)**.

In cluster Ia/b, a P-loop, suspected to bind ATP [66], with consensus sequence (K/R)GG(K/R)G is present. In cluster II, the aligned loop sequence is two residues longer. Most members of this cluster have several positively-charged residues in this loop region, but only one of the four glycines in the cluster Ia/b P-loop is somewhat conserved. Sequence logos for both loop regions are shown in Figure 6. The sequence difference at position 258 (described above) is entirely correlated with sequence and length of the loop from 161-165 for all the archaea studied. In this regard, the divergent sequences TK0431 and TGAM_1266-7 are members of cluster I.

At this time, only a few gene products in the PurP-like family have been experimentally characterized. *M. jannaschii *MJ0136, in cluster Ia, is the formate-dependent AICAR formyltransferase originally designated PurP [8]. *Pyrococcus furiosus *PF1517, in cluster Ib, and PF0421, in cluster II, have been reported to have no AICAR formyltransferase activity previously [66], although activity under different conditions or with different substrates cannot be ruled out. Crude cell extracts of *S. solfataricus *have been demonstrated to incorporate labeled formate in the conversion of ZMP to IMP [28], suggesting that a PurP-like activity is present in this organism, although we cannot identify the specific enzyme responsible from these experiments.

To establish that both cluster Ib and cluster II *purP*-like genes are involved in purine biosynthesis, we considered the identities of nearby genes. The "extra" *T. kodakaraensis purP *gene (TK0431) in cluster I is adjacent to an apparent IMP cyclohydrolase gene (*purO*) and SAICAR synthase gene (*purC*), suggesting that TK0431 is involved in purine biosynthesis as well, despite low similarity to other genes in cluster I, and the apparent redundancy of having two *purP*-like genes from *T. kodakaraensis *in cluster I. The cluster Ib and II genes in the Sulfolobales are adjacent and share a translation start site with the *purB *gene. Additional gene clusters also support assignment of both *purP*-like genes to purine biosynthesis. The *A. fulgidus *cluster II *purP*-like gene (AF0256) is clustered with *guaA1 *(not shown), while the cluster Ib gene has no apparent linkage to purine biosynthesis. *Pyrococcus abyssi *and *P. furiosus*' *purP*-like genes are both near other purine biosynthesis genes. In *Pyrobaculum islandicum*, both *purP*-like genes are adjacent to each other and also close to the large cluster of purine biosynthesis genes. Based on the available genomic data and sparse experimental data, it is reasonable to expect that both PurP-like proteins are associated with purine biosynthesis, although it is premature to draw any conclusions about the specific function of the proteins outside of cluster Ia at this time.

Although *K. cryptofilum *has been previously described as having no purine biosynthesis pathway [14], we found two adjacent *purP*-like genes, corresponding to cluster Ib and cluster II, similar to the arrangement seen in the Crenarchaeota. *K. cryptofilum *lacks other purine biosynthesis genes (along with histidine biosynthesis genes), but it could be obtaining AICAR through some unidentified salvage pathway or from a component in the environment. This organism also lacks evidence for an IMP cyclohydrolase, similar to the situation with the Crenarchaeota. We did find genes for a full set of enzymes for the conversion of IMP to GMP and AMP (data not shown), suggesting that the organism may be capable of conversion of AICAR into GMP and AMP.

Genes encoding PurO, the archaeal IMP cyclohydrolase, are largely limited to a subset of the methanogens and the Halobacteria. We inspected a sequence alignment of 25 putative PurO enzymes, especially at the positions identified by Kang and colleagues [62] as being important for contacts to the substrate. Twelve of the 13 substrate-contacting residues are fully conserved in all of the putative PurO protein sequences, while residue 54 is conserved in 23 of 25 sequences, with the two Methanocellales having a serine in place of the consensus asparagine. The consensus phylogenetic tree for PurO (Additional File 2) reproduced the order-level groupings for the organisms, although bootstrap values are low near the center of the tree. The pattern observed is consistent with either the invention of *purO *in the last euryarchaeal common ancestor followed by extensive loss early in the divergence of the Euryarchaea, or by later invention of *purO *followed by several horizontal gene transfers between euryarchaeal ancestors.

The Pyrococci, some Thermococci, and all of the Crenarchaea studied do not have an identifiable *purO *or *purH2 *gene. In the case of *Sulfolobus solfataricus*, it has been previously demonstrated that AICAR to IMP activity is present in cell extracts [28], thus if one of the two PurP-like proteins is a functional formate-dependent AICAR formyltransferase, an unidentified IMP cyclohydrolase should be present. There is some correspondence between the organisms having duplicate *purP*-like genes and those lacking a *purO *or *purH2 *gene, suggesting that one of the two PurP-like proteins might in fact catalyze the IMP cyclohydrolase reaction, but the phyletic pattern is imperfect and thus far this speculation has not yielded to experimental testing.

Unexpectedly, we identified *purO*-like genes in *Thermococcus kodakaraensis *and *T. gammatolerans*. No *purO*-like genes were found, however, in *Thermococcus sibiricus *or in any of the *Pyrococcus *species examined. Thus, in these seven Thermococcales, there are two closely related organisms with an apparent PurO, four apparently purine-biosynthesizing organisms (three Pyrococci and *T. sibiricus*) with no evidence of any known IMP cyclohydrolase gene, and one organism not performing purine biosynthesis. We considered the possibility that the PurO-like protein might be non-functional or have an alternate function in *T. kodakaraensis *and *T. gammatolerans*, as the absence of PurO in closest neighbors might suggest that some unidentified IMP cyclohydrolase is functioning in this role instead. In both *T. kodakaraensis *and *T. gammatolerans*, the unexpected *purO*-like gene is next to the rather divergent cluster I *purP*-like gene (discussed above) and a second copy of a *purC-*like gene. The ancestor of these two Thermococci may have acquired this cluster of "extra" genes via horizontal gene transfer. Supporting this hypothesis, *T. kodakaraensis' purO *and extra *purP *are within a cluster of atypical genes according to Cortez *et al*. [67] and do not match the mode for codon usage [68] in *T. kodakaraensis*. An evolutionary advantage to having this extra gene cluster is not readily apparent, although it may be that this PurO-like protein is a more efficient IMP cyclohydrolase than the unidentified IMP cyclohydrolase that we suspect is present in the Pyrococci and the Crenarchaeota. Activity of the *T. kodakaraensis *PurO (TK0430) has recently been experimentally confirmed *in vitro *[C. A. Hunter, N. I. Plymale, and C. A. Sarisky, unpublished results].

Part of the difficulty in detecting a gene encoding IMP cyclohydrolase activity on the basis of genome gazing is that the IMP cyclohydrolase enzyme has a relatively modest role to play in the catalysis. It only needs to hold its substrate in the appropriate orientation for intramolecular nucleophilic attack and perhaps provide some general acid/base-capable side chains in the vicinity. Thus, almost any enzyme with a nucleotide monophosphate binding site is a potential IMP cyclohydrolase. One of us (CAS) is pursuing a knockout-complementation approach to identify the missing IMP cyclohydrolase, as well as experimental characterization of proteins encoded by *purP*-like genes from both clusters.

## Conclusions

Although we were able to identify a candidate gene for most of the necessary biosynthetic steps in the purine biosynthesis pathway in most of the organisms studied, a number of questions remain. An IMP cyclohydrolase enzyme has not been identified for any of the Crenarchaeota nor for a subset of the Thermococcales. A GAR formyltransferase is similarly missing in seven of the Euryarchaeota studied.

A number of duplicate genes (especially *purF *and *purP*) have been identified, but a biochemical or regulatory explanation for these duplications has not been established. Widespread duplication implies some selective advantage, particularly when it appears in organisms with relatively small genomes. Although an isolated case of duplication in closely related species (such as *purN *in *M. barkeri *and *M. acetivorans*) may be a random occurrence, gene duplications that occur across a range of less-related species merit further investigation, as they presumably serve a specific function in the organisms that have retained them.

We generally observed the same pattern of gene distribution across sequenced organisms in the same order, but more distantly related organisms exhibit phylogenetic patterns not entirely consistent with accepted archaeal phylogeny. For instance, we observe orders within both the Euryarchaeota and the Crenarchaeota that have *purT *and others orders that use *purN*. Likewise, within the Euryarchaeota, we have orders that have *purO *and *purP*, bifunctional *purH*, a single domains of *purH *with either *purO *or *purP*, and those that have *purP *but neither *purH2 *nor *purO*. If we accept that the archaeal phylogeny is largely correct, the implication of this distribution pattern is that the last archaeal common ancestor contained a complete purine biosynthesis pathway, with numerous gene loss or gene transfer events occurring. The distribution of genes is largely consistent with Makarova and coworkers' conservative estimate of the gene content of the last archaeal common ancestor [69], although we suspect that the last archaeal common ancestor had at least one GAR formyltransferase (PurN and/or PurT), with patchy loss or gain to produce the pattern presently observed in the Crenarchaeota and Euryarchaeota.

Finally, we must ask: Can humans improve upon automated annotations? As shown in Table 1, the majority of gene product names and E. C. numbers assigned in IMG for predicted proteins involved in purine biosynthesis are correct, despite many having been assigned by automated or semi-automated processes. However, there is room for improvement, especially in the case of multiple-domain proteins, which were susceptible to under-annotation, with the protein assigned the name and E. C. number for only a single domain, and over-annotation, with some proteins assigned names and E. C. numbers for domains that were not present.

We also detected problems with names and E. C. numbers assigned to predicted proteins that were similar to known enzymes but that lacked the active site residues believed to be required for function. These annotations were especially suspect in organisms containing two candidates for a particular enzyme, with both candidates typically being assigned the same enzyme name. Assignment of a highly specific enzyme name to a predicted protein that neither contains the expected active site nor is the bidirectional best hit for that enzyme is questionable at best. Assignment of specific functions based on low quality evidence carries the risk that future annotations will propagate that name to an entire subclass of proteins without the named activity. We believe that the subsystems approach [12], with expert annotators (whether computers or humans) working on specific pathways across a number of genomes can substantially improve the quality of gene annotations.

## Methods

### Organisms

Fully sequenced, non-draft archaeal genomes available in IMG (as of May 2010) were considered. Organisms studied are listed in Tables 1 and 2. Gene codes used in Additional File 1 and throughout the paper are the locus tags available through IMG [70]. The analysis was further limited to the single *M. maripaludis, Sulfolobus islandicus*, and *S. solfataricus *cultivars listed in Figures 2, 3 and Additional File 1.

### Identification of gene candidates

Except where otherwise noted, gene candidates were identified using the BLAST search tool provided through the Joint Genome Institute's IMG site http://img.jgi.doe.gov, with results limited to the selected fully-sequenced archaea. Known purine biosynthesis enzymes from *E. coli, B. subtilis*, or characterized archaeal enzymes (such as *M. jannaschii *PurP and PurO) were used as input sequences for the BLAST searches. Where the archaeal sequences were only distantly related to the starting enzyme sequences, sequential BLAST searching was performed to identify additional gene candidates. Sequence alignments produced with IMG's built-in Clustal tool or with ClustalX, version 2.0.12 [71], were inspected manually for added or missing domains and conserved residues.

The DNA sequences flanking the *purF*-like genes Mthe_0230 and CENSYa_1910 were retrieved from the UCSC Archaeal Genome Browser [72] and translated after problems with the predicted protein sequence available through both this source and IMG were detected.

### Phylogenetic trees

The amino acid sequence of MJ0136 was used to identify PurP-like sequences using IMG's BLAST tool, as described above. Sequences were aligned with ClustalX, version 2.0.12 [71], followed by removal of regions of low sequence conservation and dubious alignment using SeaView [73]. Phylogenetic trees were generated from the aligned sequences using PhyML [74,75] with the JTT substitution model, an estimated proportion of invariable sites, and an estimated gamma distribution parameter. Bootstrapping was performed using 100 data sets, generated with the same parameters. Phylogenetic trees were drawn from the PhyML output using Dendroscope [76]. Phylogenetic trees are provided in Additional File 2. Sequence logos were created with WebLogo [77].

### Error analysis

We evaluated the "Gene Product Name" as exported from IMG's gene cart and any E. C. number exported as part of the gene product name for discrepancies between these assignments and our manual curation. We classified a Gene Product Name as erroneous when it clearly applied to a different enzymatic reaction. Minor typographical errors were not considered errors in this analysis. Gene symbols were evaluated when they were not blank and did not duplicate the locus tags. Because it was not possible to distinguish between typographical errors and true errors in the gene symbol field or E.C. number, any discrepancy in these fields was counted as an error. Other fields containing enzyme names and E. C. numbers available in IMG were not considered in the error analysis. Additional details of the error analysis are available in Table 3.

## Competing interests

The authors declare that they have no competing interests.

## Authors' contributions

CAS, AMB, and SLH performed initial identification and curation of genes. CAS identified and curated additional genes, analyzed results, and drafted the manuscript. RHW contributed to the analysis. AMB and RHW critically revised the manuscript for grammar and content. All authors read and approved the final manuscript.

## Reviewer comments

Reviewer #1: Dr. Céline Brochier-Armanet, Aix-Marseille Université, France

In their paper, Brown et al. present an exhaustive and in-depth investigation of the purine biosynthesis pathway in Archaea. Starting from experimentally-characterised enzymes they identified homologues in 65 archaeal genomes using best-reciprocal blast hits and a "guilty by association" strategy. The authors show that 58 archaea harbour a nearly complete purine biosynthesis pathway suggesting that only slight variations occurred from a canonical pathway that was present in the ancestor of Archaea. Moreover, they highlight the absence of important enzymes in some genomes harbouring a nearly complete purine biosynthesis pathway suggesting that some enzymes have been replaced in some genomes by non-homologous enzymes or that some steps of the pathway are bypassed. Based on this analysis the authors have re-annotated some genes and identified new putative enzymes.

I think that this contribution is of great interest to the community working on the archaea and more generally to microbiologists. However, I have a few general comments on the manuscript. My main concern is that the paper is not easy to read because of the important amount of information/data presented. Unfortunately this may discourage non-specialist readers despite the interest of the subject.

For instance, the paper would have benefited from a longer introduction presenting current knowledge on purine biosynthesis in the three domains of life and its connection with histidine biosynthesis. In the current version of the manuscript the pathway is only presented progressively along the result/discussion section. In fact, without minimal starting information about the pathway the reading of the results section is very hard. Moreover a brief overview of the archaeal phylogeny (illustrated by a reference phylogenetic tree) must be provided to the readers that are not familiar with the archaeal domain (Tables 1 and 2 are not sufficient).

### Author's response

*We thank the reviewer for these helpful comments. We have expanded the introduction, hopefully improving the accessibility of the manuscript to non-specialist readers. We have also added a taxonomy-based phylogenetic tree to *Figures 2, 3, *to give the reader some indication of the relationships between these organisms*.

Second, I think that systematic phylogenetic investigation of each enzyme would have been of great interest. Indeed, if the presence of each enzyme in most archaeal genomes may suggest a vertical inheritance of this pathway from the last common ancestor of this domain, it would have been interesting to have a better picture of the duplication, loss and horizontal gene transfer events that have affected the evolutionary history of these genes. For instance, this would allow discriminating between the two types of purE, determining if purO and purP have been co-transferred with purC in the two thermococci that harbour two copies of purC, determining precisely the origin of purU in Halobacteriales and Thaumarchaeota, etc. Therefore I encourage the authors to provide phylogenetic trees of the mainly discussed enzymes as figures and to provide other phylogenetic trees as supplementary material. This would provide a more precise picture of the evolution of this pathway and greatly increase our knowledge of archaeal evolution.

### Author's response

*We have added phylogenetic trees for all proteins in *Additional File 2, *along with some further discussion of these trees in the manuscript body*.

Third, the paper lacks of a few illustrations summarizing the main results. For instance, the authors should map the identified enzymes on a reference archaeal phylogeny in order to provide a synthetic overview of their results. Similarly, a figure showing the genomic organisation of genes involved in purine biosynthesis would surely be appreciated by the reader (see for instance (Desmond et al. 2007)).

### Author's response

*We have made the recommended addition of a phylogenetic tree to *Figures 2, 3. *We have not produced figures showing genomic organization due to the difficulty of showing up to 18 genes (many not located in operons) for 65 organisms in any printable format*.

Reviewer #2: Dr. Kira S. Makarova, NIH, NLM (nominated by Dr Eugene Koonin, NIH, NLM)

This paper presents a detailed description of proteins involved or thought to be involved in purine biosynthesis in archaea. After reading the paper I have an impression that this is meant to be a guideline for annotation of these proteins in archaeal genomes. This guideline comes from one of the most competent group of researchers in the field of archaeal biochemistry and therefore their description of these proteins is absolutely accurate and the paper will be very helpful for correction of automatic pipelines for protein annotation. This being said I would rather suggest the authors instead of writing a separate paper for a particular pathway to develop and maintain a database of the enzymes involved or implicated in archaeal central metabolism (and publish it in a NAR database issue). Such a database could be directly linked to the annotation pipelines and considerably improve the future genome annotation or reannotation of the previously submitted genomes.

### Author's response

*We thank the reviewer for her kind comments about our accuracy and for additional very helpful comments that have improved this manuscript. A database focused on archaeal metabolic pathways is an interesting alternative, although a database just for purines strikes us a bit too specialized to be generally useful. To some extent, the proposed database would duplicate the information already available, such as in SEED and NMPDR. One of us (CAS) is working to get our new and corrected annotations into IMG*.

I do not find useful the report about the number of errors in the enzyme annotation - it is a well-known problem. As the understanding, that the best annotations are produced manually by experts. On the other hand it is quite hard to find a formal approach to analyze and compare scientifically the number of errors since there is no common rules for genomes annotation, no widely-accepted "golden standard" to compare with and the fact that even the results of manual annotation still depend on the parameters of sequence similarity detection procedure and a database content, not even mentioning the constantly changing status of the knowledge in the field. Thus in this respect the whole part of the paper concerning these errors can be only considered as an opinion.

### Author's response

*We agree that there is some opinion in determining what is an error in the absence of experimental characterization. We have added more explicit information to the manuscript about what we counted as an error. Even in the presence of experimental characterization, there is the question of what to do with negative results. Is the enzyme inactive because it is being tested for the wrong function, or simply because it needs another protein partner to exhibit measurable activity? We believe that the presence of obvious errors in a major database on a "well-understood" pathway merits further discussion, even if differences in error calling will of course result in different percentages*.

I also had a hard time to understand from the abstract what was exactly the focus (if any) of the paper or do authors believe that they report results of any original research (if so what it is?). Since I consider this paper as an annotation guideline I am hesitant to ask authors to justify scientifically (meaning the reconstruction of phylogenetic trees for all the genes in question and comparison of maximum likelihood estimates of alternative evolutionary scenarios) some conclusions from the abstract like, for example: "The patchy distribution of purine biosynthesis in archaea is consistent with a pathway that has been shaped by horizontal transfer, duplication, and gene loss", which I would do for a paper reporting an original research result.

### Author's response

*As described above, we have provided more information on the phylogeny of these proteins as *Additional File 2*. We hope you will agree that careful (re-)annotation of a pathway of this size and with this level of variability is a substantial undertaking. This work is, indeed, more descriptive than most manuscripts. We hope it will serve the community by identifying parts of the pathway that merit experimental characterization and also enzymes whose annotation is not well-supported and thus might be reasonably tested for novel functions for which an enzyme candidate has not been proposed*.

Reviewer #3: Dr. Michael Galperin, National Center for Biotechnology Information, NIH

This reviewer provided no comments for publication.

## Acknowledgements and funding

CAS was supported by a grant from the Jeffress Memorial Trust, J-870, and by a Cottrell Single Investigator Research Award, 19885. AMB and SLS received summer support from a Roanoke College Bondurant Scholarship. RHW was supported by a National Science Foundation grant, MCB-0231319.

## Supplementary Material

Additional file 1 **Spreadsheet with gene locus tags for all purine biosynthesizing genes identified**. This Excel spreadsheet contains full organism names and taxonomies, IMG taxon IDs, and gene locus tags for all purine biosynthesizing genes identified, corresponding to the locus tags available in IMG. Locus tags are in parentheses when significant features (such as expected active site residues or expected domains) are absent from the predicted protein. The designation "PurH1" is used for the typically C-terminal AICAR formyltransferase domain, while the designation "PurH2" is used for the typically N-terminal IMP cyclohydrolase domain of full-length PurH. For other predicted proteins, the locus tags were separated by a + when two adjacent ORFs appeared to each encode part of full-length expected protein. The designations "(in purL)" and "(in purE)" were used to denote cases where the expected function appeared to be provided by the PurL or PurE protein, as described further in the text. Click here for file

Additional file 2 **Zip file containing additional phylogenetic trees**. A set of phylogenetic trees generated as described in the Methods section. Locus tags were used for archaeal proteins, while species names were used for non-archaeal proteins used for comparisons. Click here for file
