# Supplementary material for: Purine biosynthesis in archaea: variations on a theme
Source: Biol Direct. 2011 Dec 14;6:63. doi: 10.1186/1745-6150-6-63 (PMC3261824; doi:10.1186/1745-6150-6-63)
Supplement: Additional file 2 — Zip file containing additional phylogenetic trees. A set of phylogenetic trees generated as described in the Methods section. Locus tags were used for archaeal proteins, while species names were used for non-archaeal proteins used for comparisons. [file 1745-6150-6-63-S2.ZIP › Supplemental data file 1/Read me.docx]

Internal node labels are bootstrap values for 100 replicates. Terminal node labels are the locus tags in Supplemental Data Table 1 for the studied archaea. Where non-archaea were also used in tree building, the species name has been used as the terminal node label, with E designating a eukaryote and B designating a bacterium.

These are unrooted trees, as described in the methods section. Prior to pdf creation, trees were arbitrarily rooted near the midpoint and/or to highlight common phylogenetic groupings.
