# Supplementary figures and images for: Purine biosynthesis in archaea: variations on a theme
Source: Biol Direct. 2011 Dec 14;6:63. doi: 10.1186/1745-6150-6-63 (PMC3261824; doi:10.1186/1745-6150-6-63)

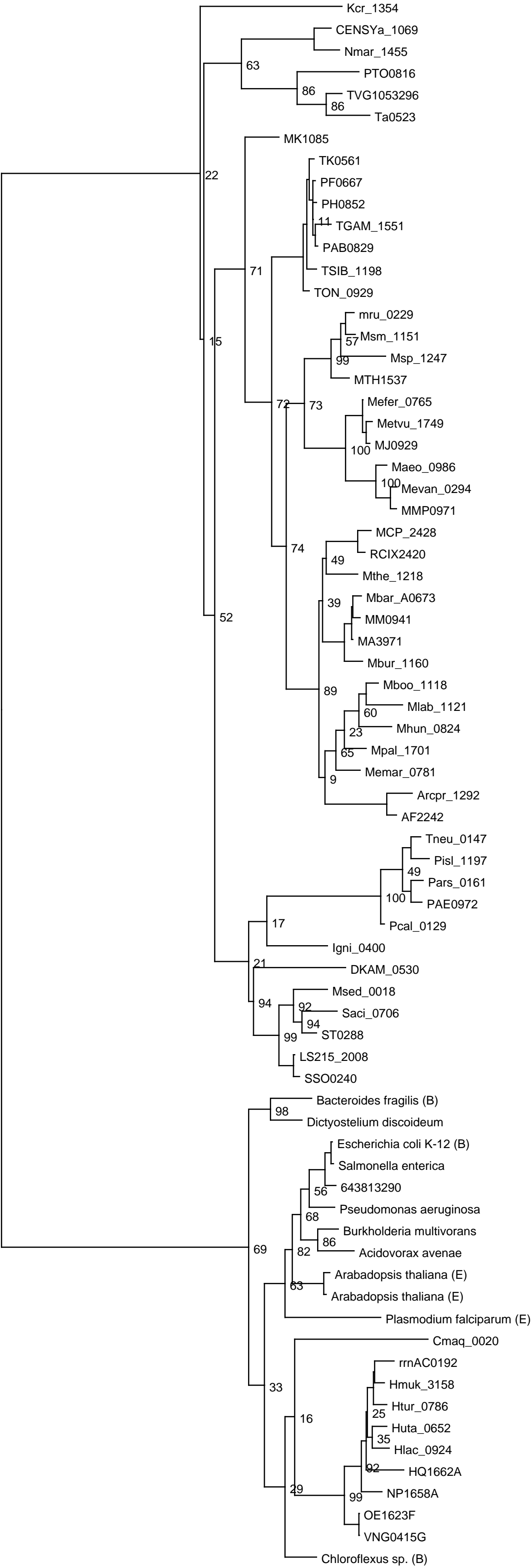

Supplement: Additional file 2 — Zip file containing additional phylogenetic trees. A set of phylogenetic trees generated as described in the Methods section. Locus tags were used for archaeal proteins, while species names were used for non-archaeal proteins used for comparisons. [file 1745-6150-6-63-S2.ZIP › Supplemental data file 1/PurB tree with non-archaea.pdf]

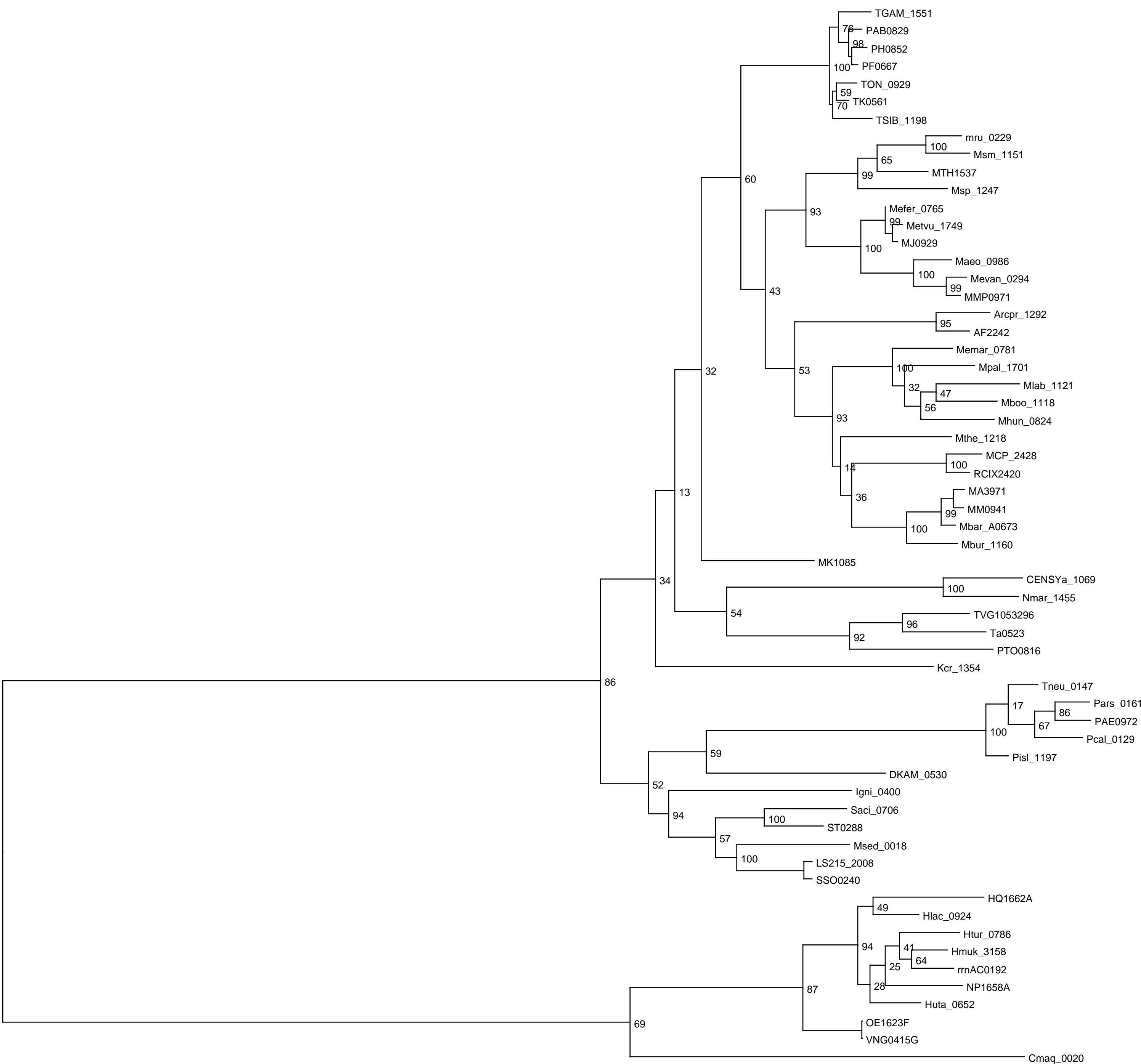

Supplement: Additional file 2 — Zip file containing additional phylogenetic trees. A set of phylogenetic trees generated as described in the Methods section. Locus tags were used for archaeal proteins, while species names were used for non-archaeal proteins used for comparisons. [file 1745-6150-6-63-S2.ZIP › Supplemental data file 1/PurB tree.pdf]

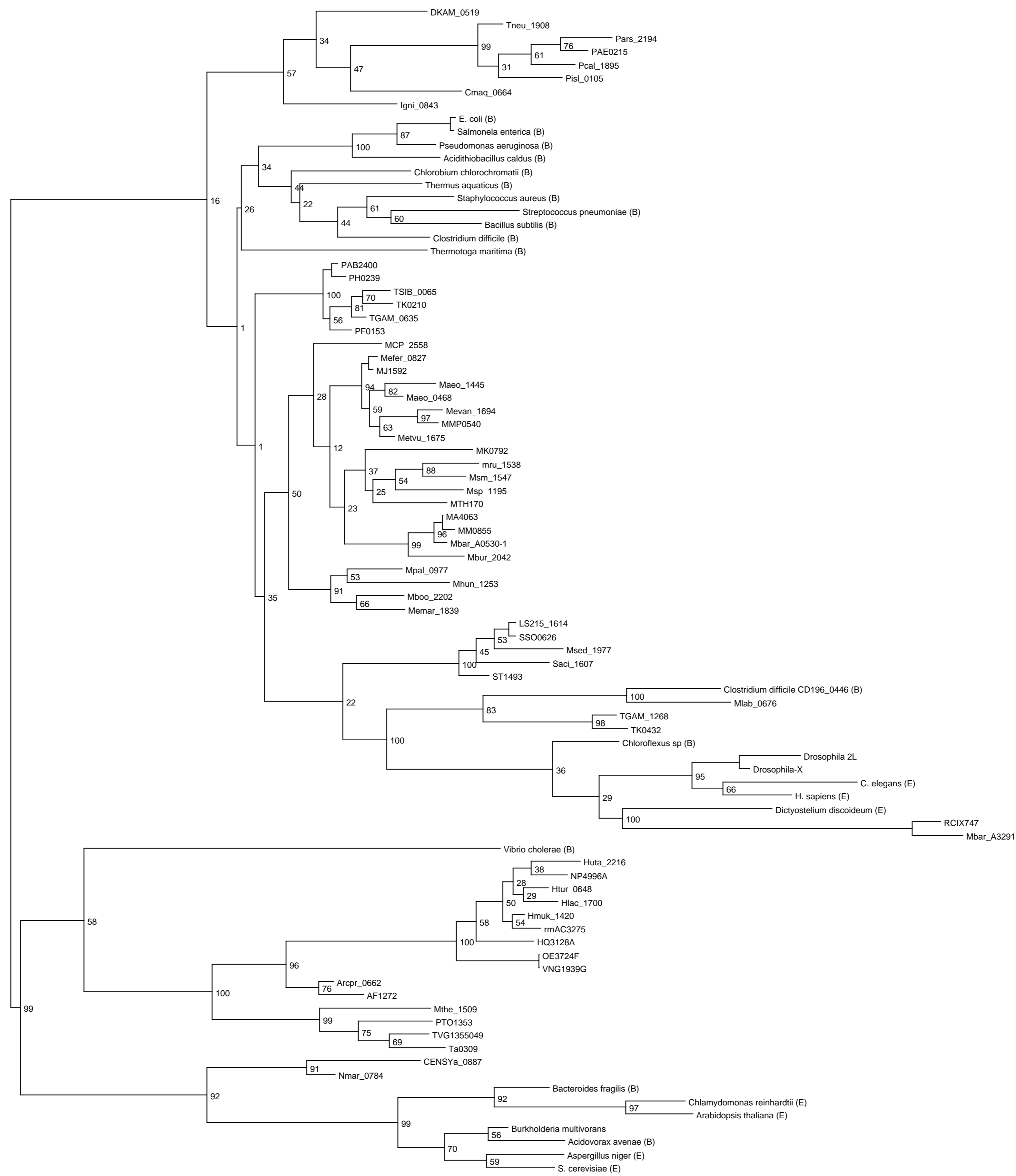

Supplement: Additional file 2 — Zip file containing additional phylogenetic trees. A set of phylogenetic trees generated as described in the Methods section. Locus tags were used for archaeal proteins, while species names were used for non-archaeal proteins used for comparisons. [file 1745-6150-6-63-S2.ZIP › Supplemental data file 1/PurC tree with non-archaea.pdf]

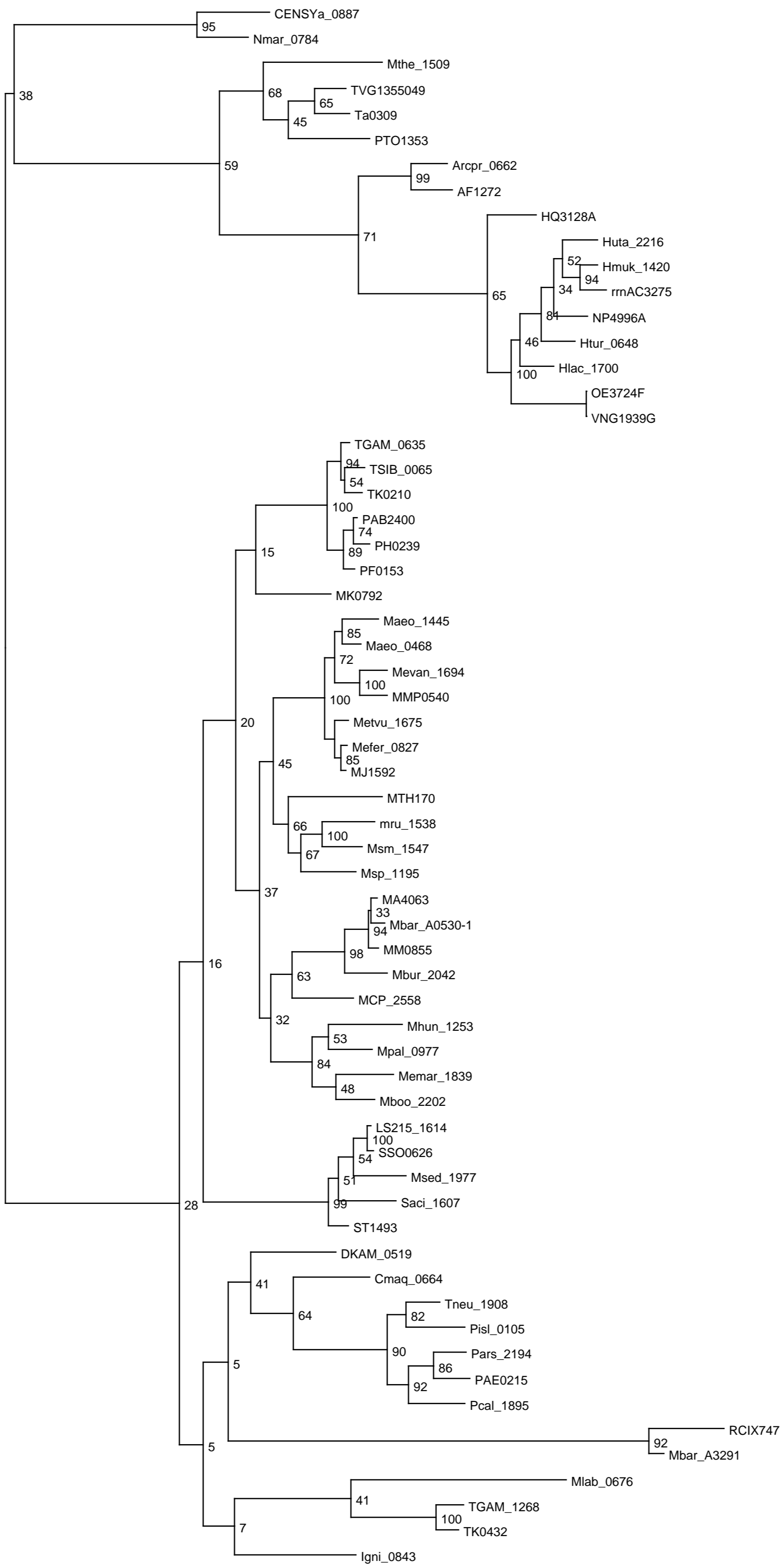

Supplement: Additional file 2 — Zip file containing additional phylogenetic trees. A set of phylogenetic trees generated as described in the Methods section. Locus tags were used for archaeal proteins, while species names were used for non-archaeal proteins used for comparisons. [file 1745-6150-6-63-S2.ZIP › Supplemental data file 1/PurC tree.pdf]

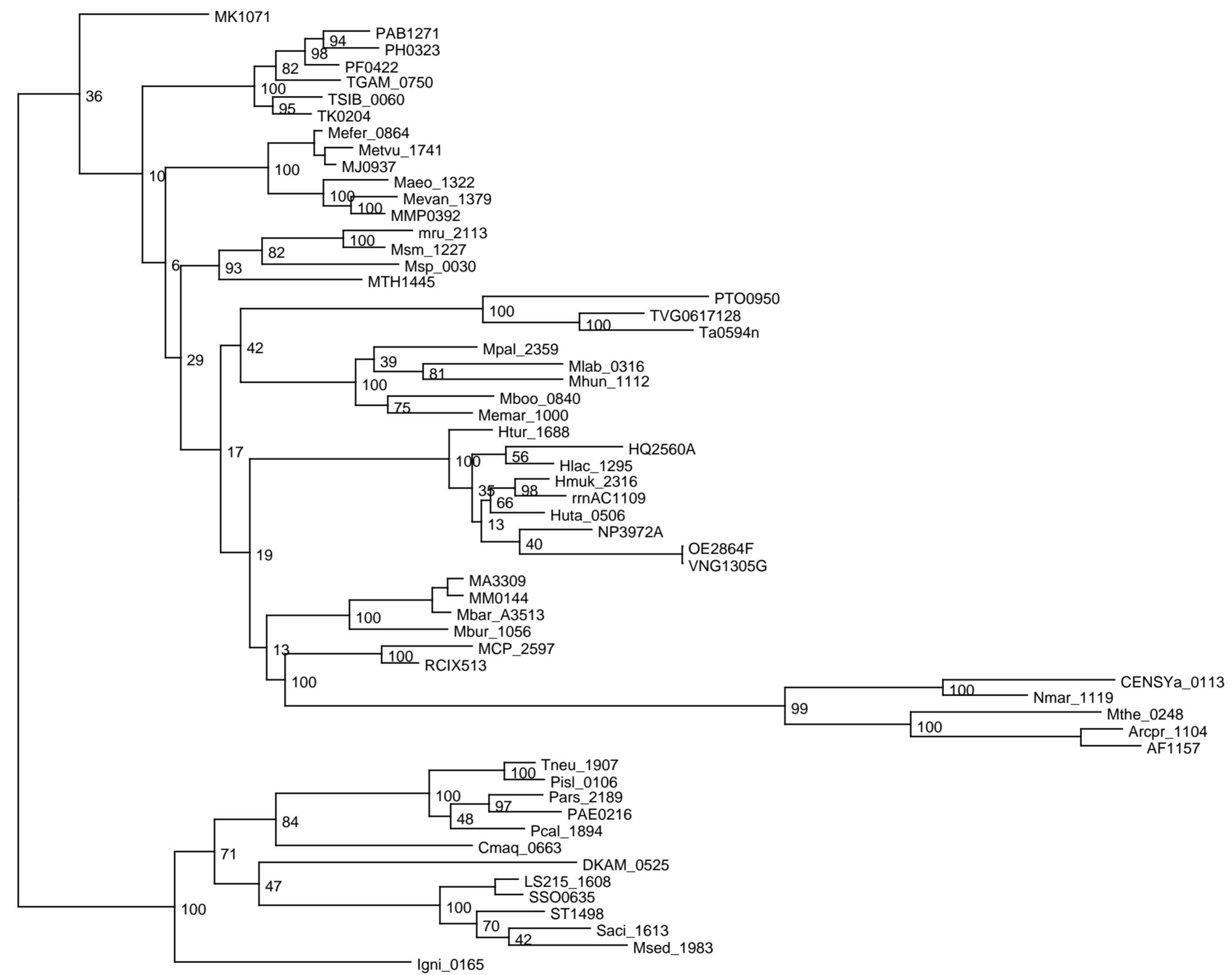

Supplement: Additional file 2 — Zip file containing additional phylogenetic trees. A set of phylogenetic trees generated as described in the Methods section. Locus tags were used for archaeal proteins, while species names were used for non-archaeal proteins used for comparisons. [file 1745-6150-6-63-S2.ZIP › Supplemental data file 1/PurD tree.pdf]

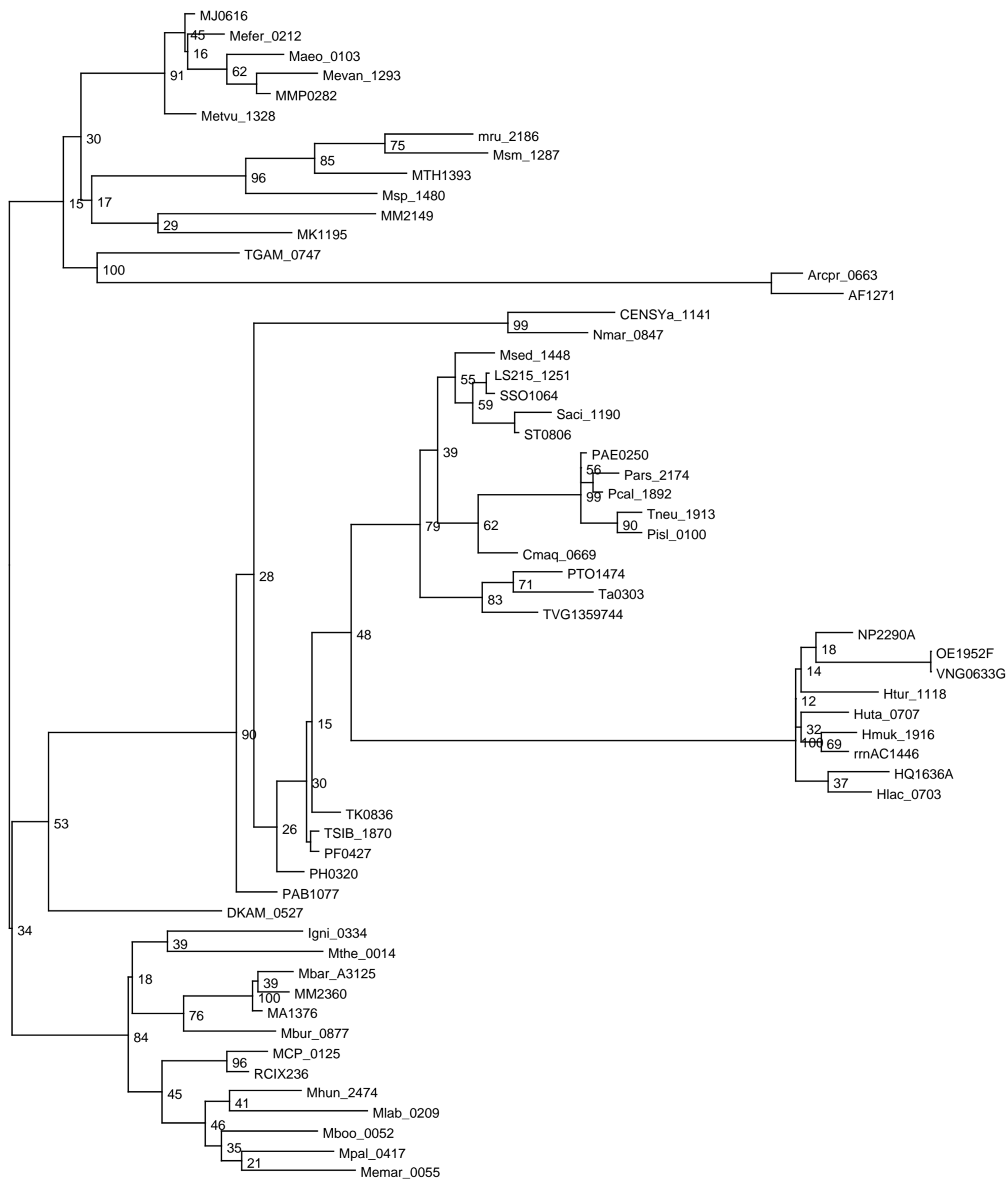

Supplement: Additional file 2 — Zip file containing additional phylogenetic trees. A set of phylogenetic trees generated as described in the Methods section. Locus tags were used for archaeal proteins, while species names were used for non-archaeal proteins used for comparisons. [file 1745-6150-6-63-S2.ZIP › Supplemental data file 1/PurE tree.pdf]

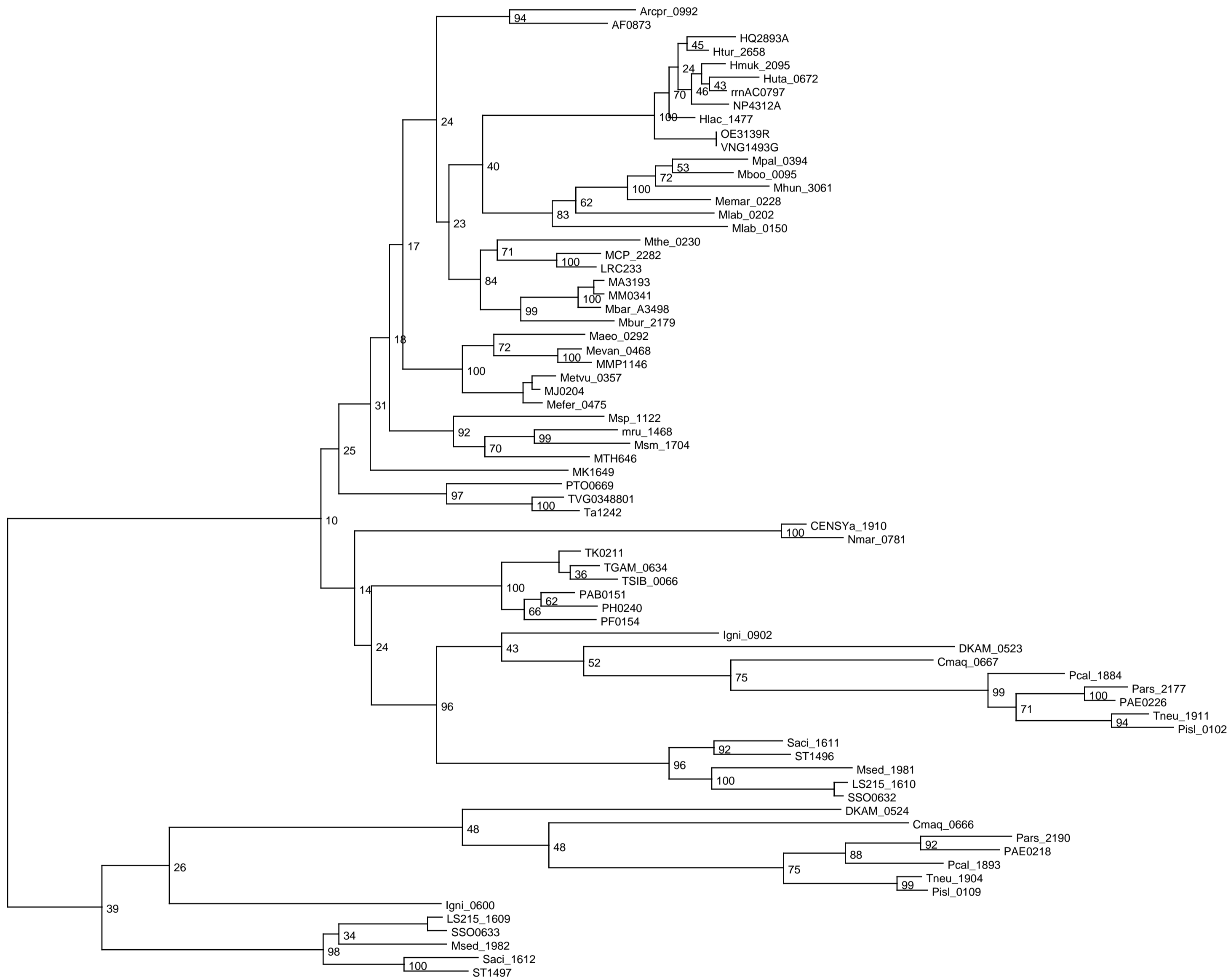

Supplement: Additional file 2 — Zip file containing additional phylogenetic trees. A set of phylogenetic trees generated as described in the Methods section. Locus tags were used for archaeal proteins, while species names were used for non-archaeal proteins used for comparisons. [file 1745-6150-6-63-S2.ZIP › Supplemental data file 1/PurF tree.pdf]

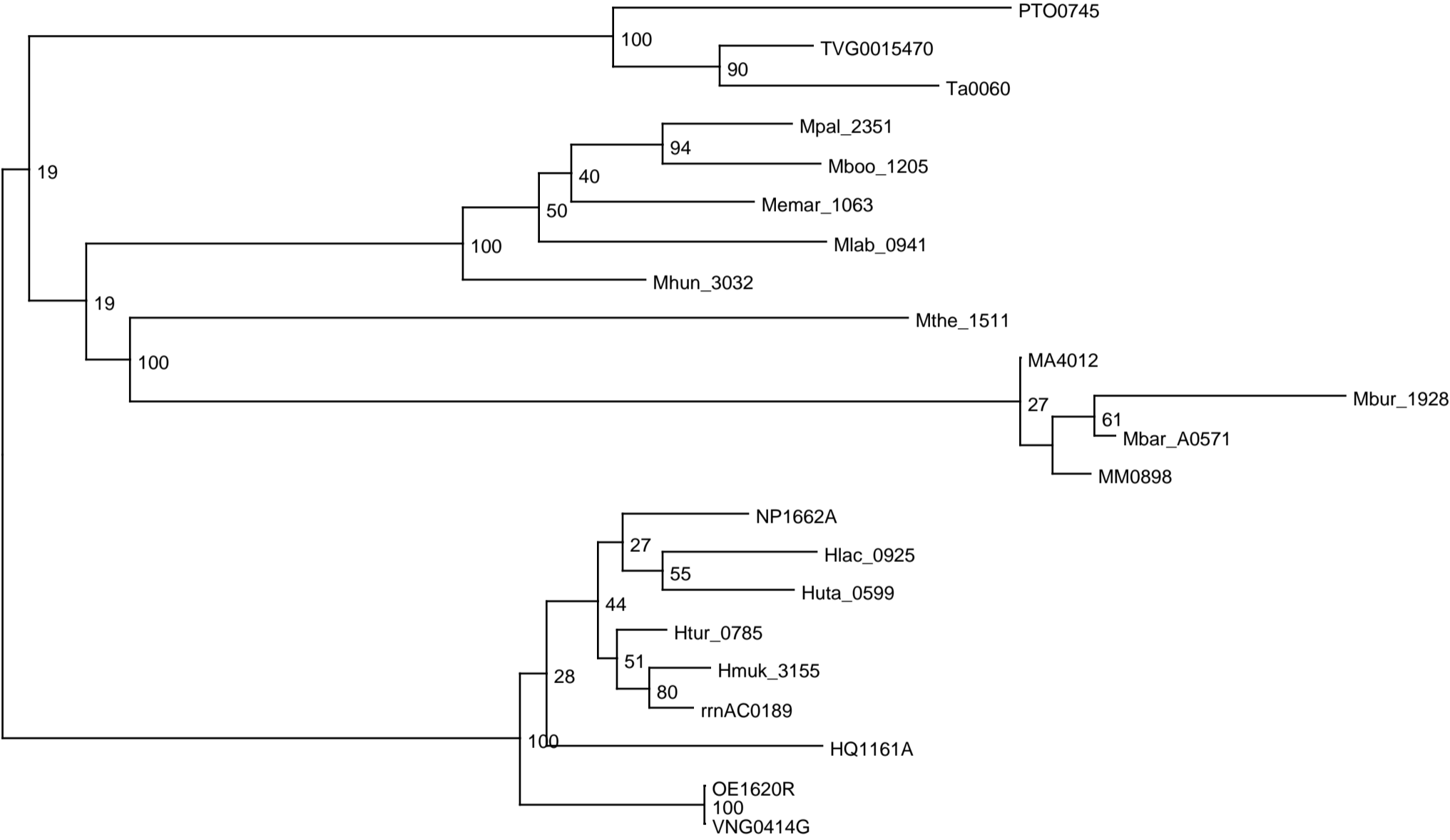

Supplement: Additional file 2 — Zip file containing additional phylogenetic trees. A set of phylogenetic trees generated as described in the Methods section. Locus tags were used for archaeal proteins, while species names were used for non-archaeal proteins used for comparisons. [file 1745-6150-6-63-S2.ZIP › Supplemental data file 1/PurH1 tree.pdf]

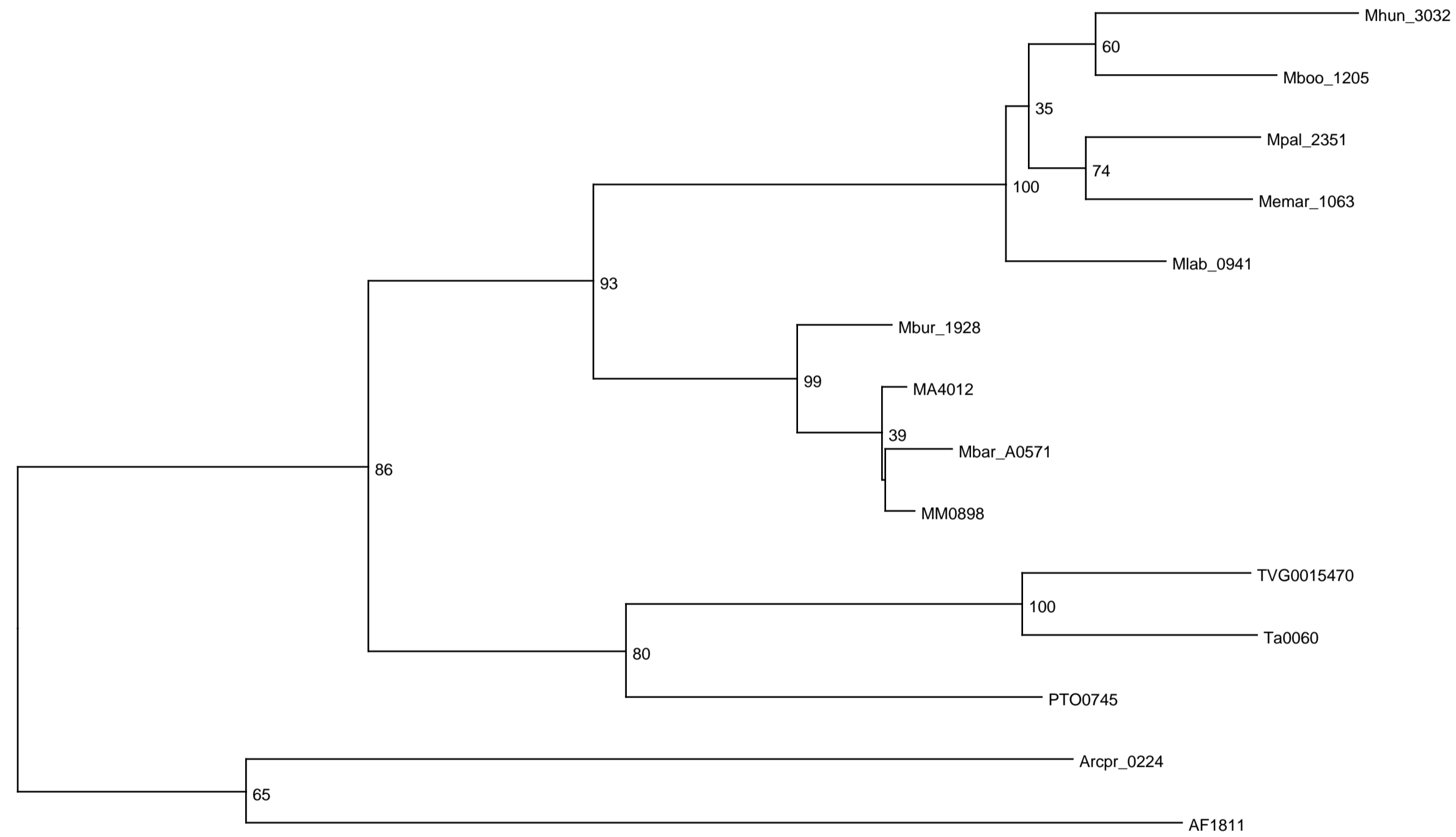

Supplement: Additional file 2 — Zip file containing additional phylogenetic trees. A set of phylogenetic trees generated as described in the Methods section. Locus tags were used for archaeal proteins, while species names were used for non-archaeal proteins used for comparisons. [file 1745-6150-6-63-S2.ZIP › Supplemental data file 1/PurH2 tree.pdf]

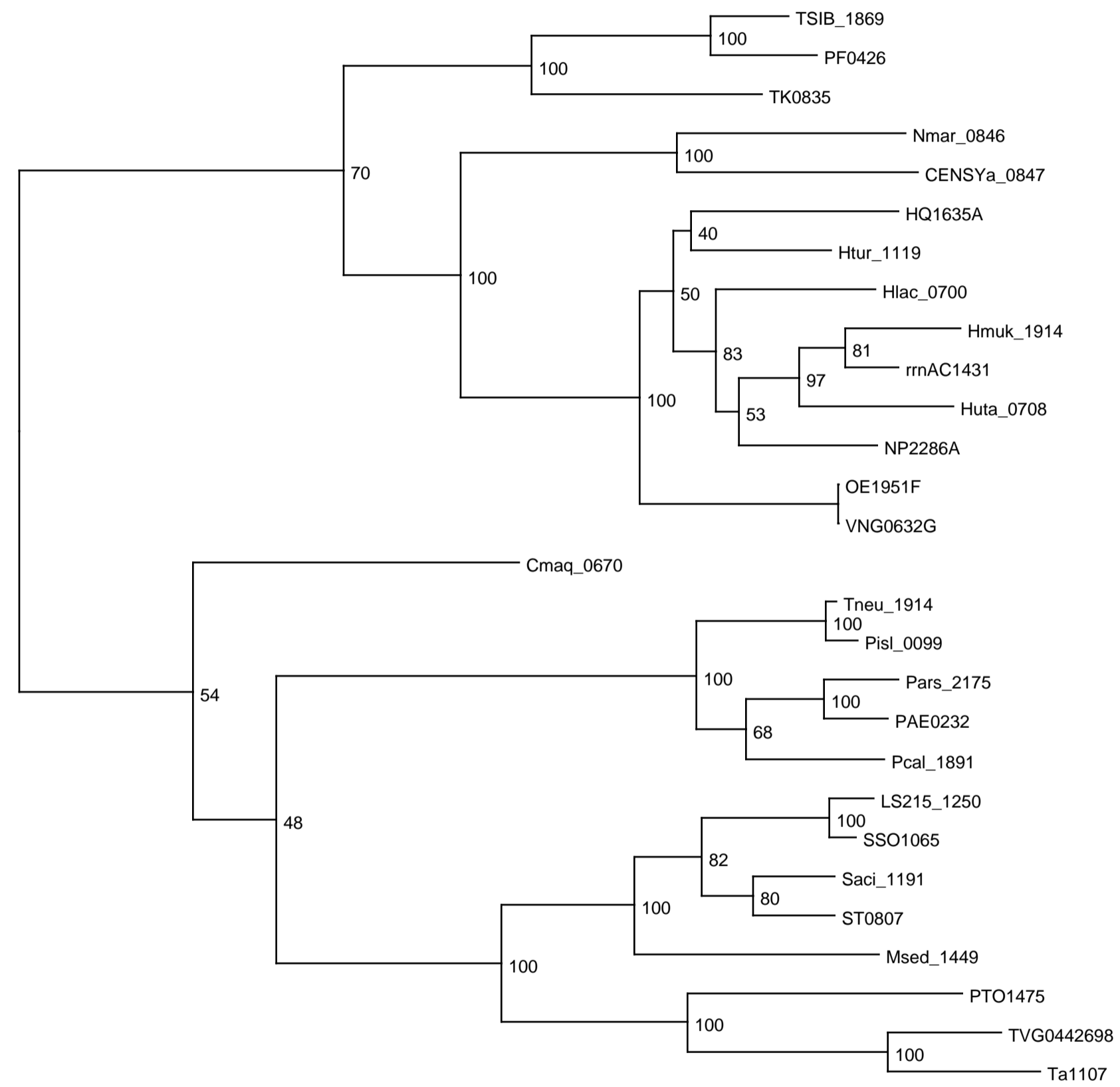

Supplement: Additional file 2 — Zip file containing additional phylogenetic trees. A set of phylogenetic trees generated as described in the Methods section. Locus tags were used for archaeal proteins, while species names were used for non-archaeal proteins used for comparisons. [file 1745-6150-6-63-S2.ZIP › Supplemental data file 1/PurK tree.pdf]

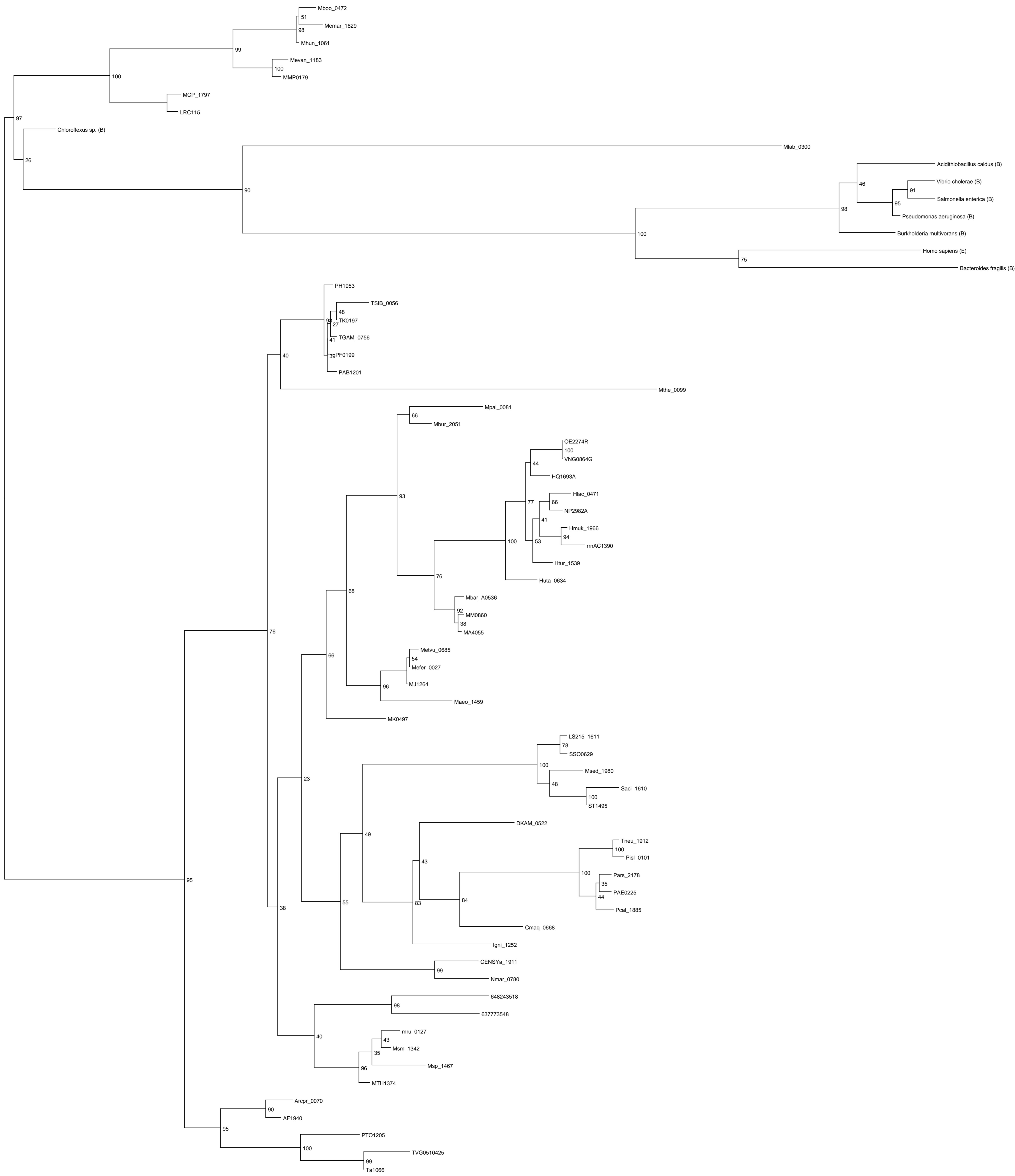

Supplement: Additional file 2 — Zip file containing additional phylogenetic trees. A set of phylogenetic trees generated as described in the Methods section. Locus tags were used for archaeal proteins, while species names were used for non-archaeal proteins used for comparisons. [file 1745-6150-6-63-S2.ZIP › Supplemental data file 1/PurL tree with non-archaea.pdf]

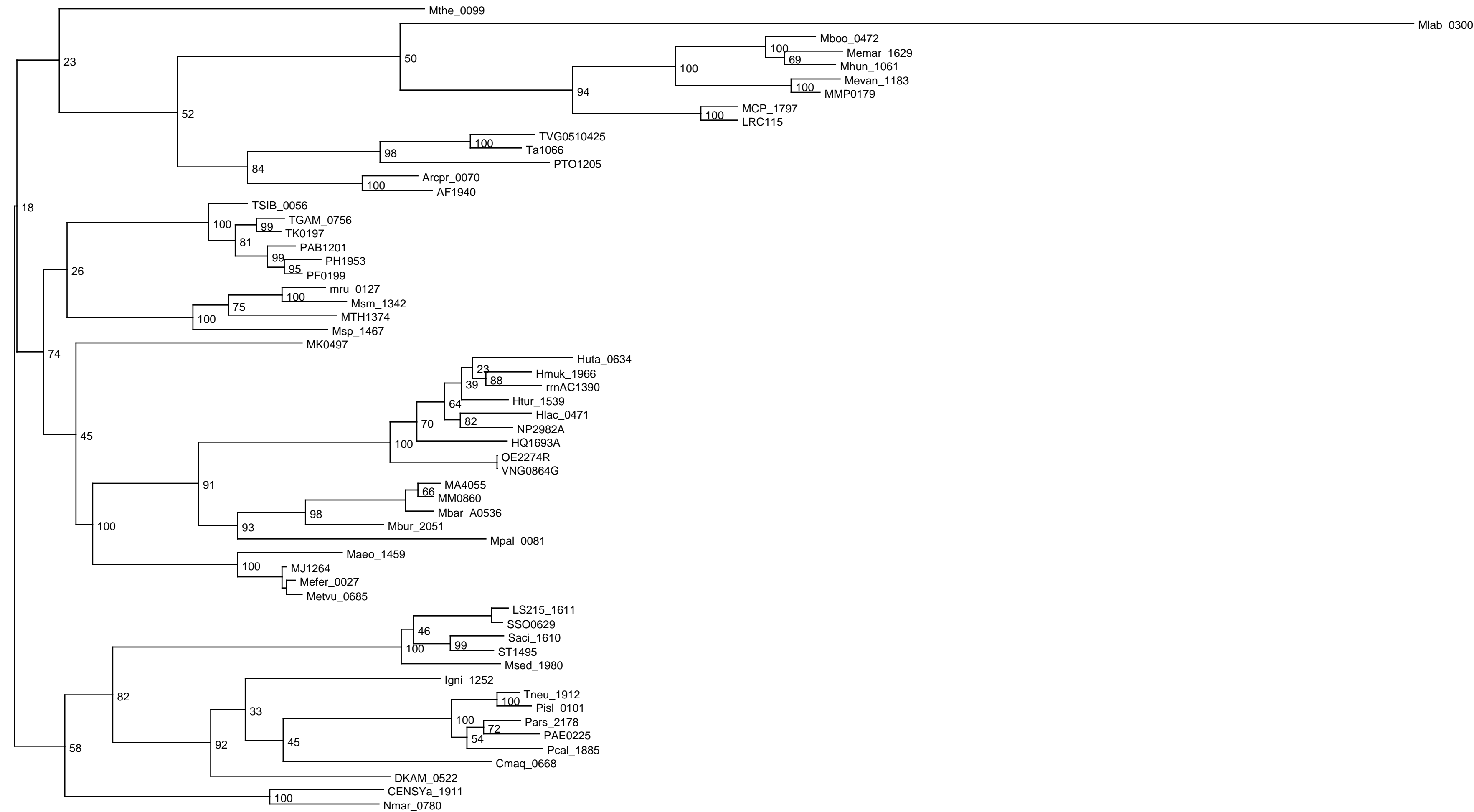

Supplement: Additional file 2 — Zip file containing additional phylogenetic trees. A set of phylogenetic trees generated as described in the Methods section. Locus tags were used for archaeal proteins, while species names were used for non-archaeal proteins used for comparisons. [file 1745-6150-6-63-S2.ZIP › Supplemental data file 1/PurL tree.pdf]

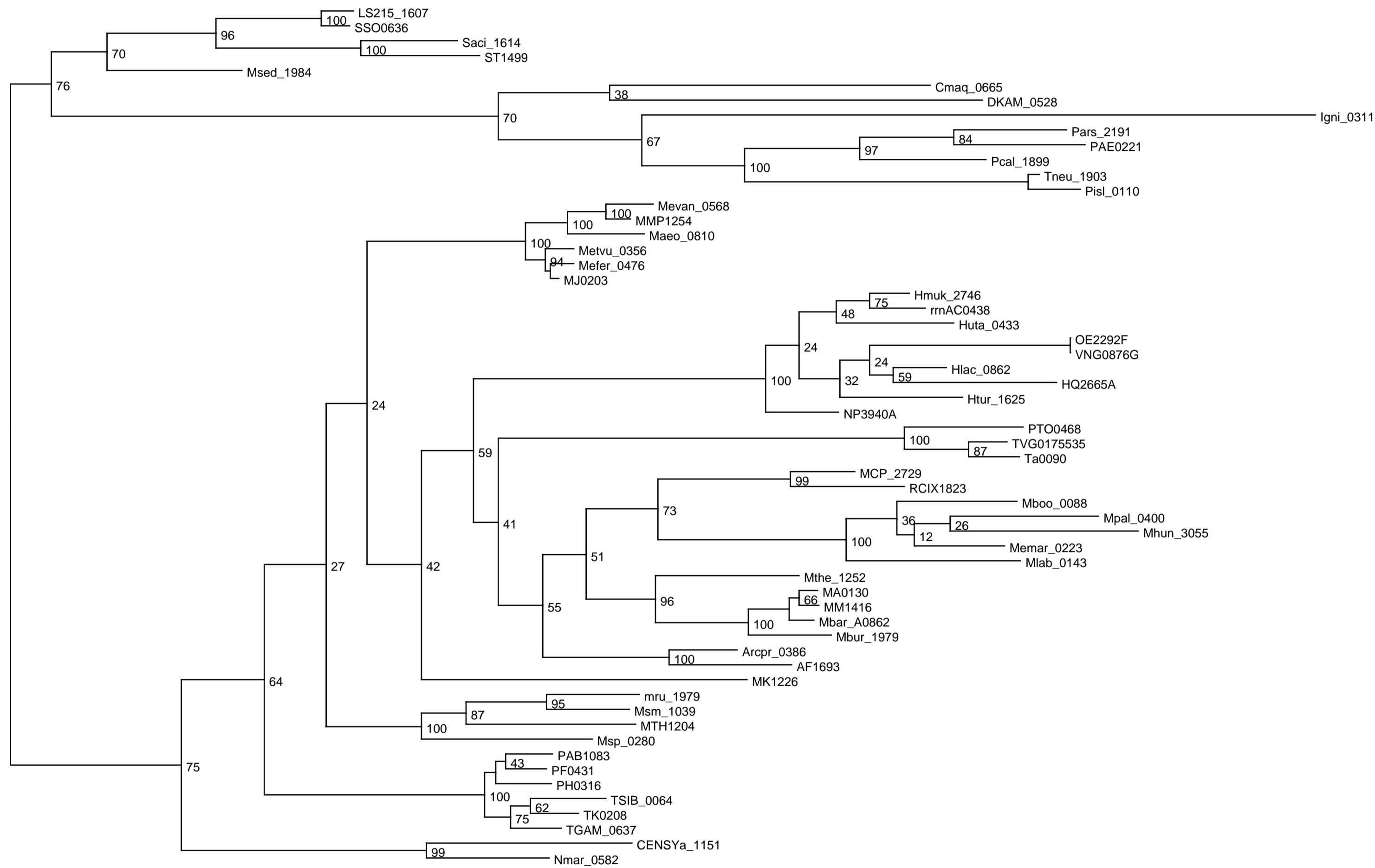

Supplement: Additional file 2 — Zip file containing additional phylogenetic trees. A set of phylogenetic trees generated as described in the Methods section. Locus tags were used for archaeal proteins, while species names were used for non-archaeal proteins used for comparisons. [file 1745-6150-6-63-S2.ZIP › Supplemental data file 1/PurM tree.pdf]

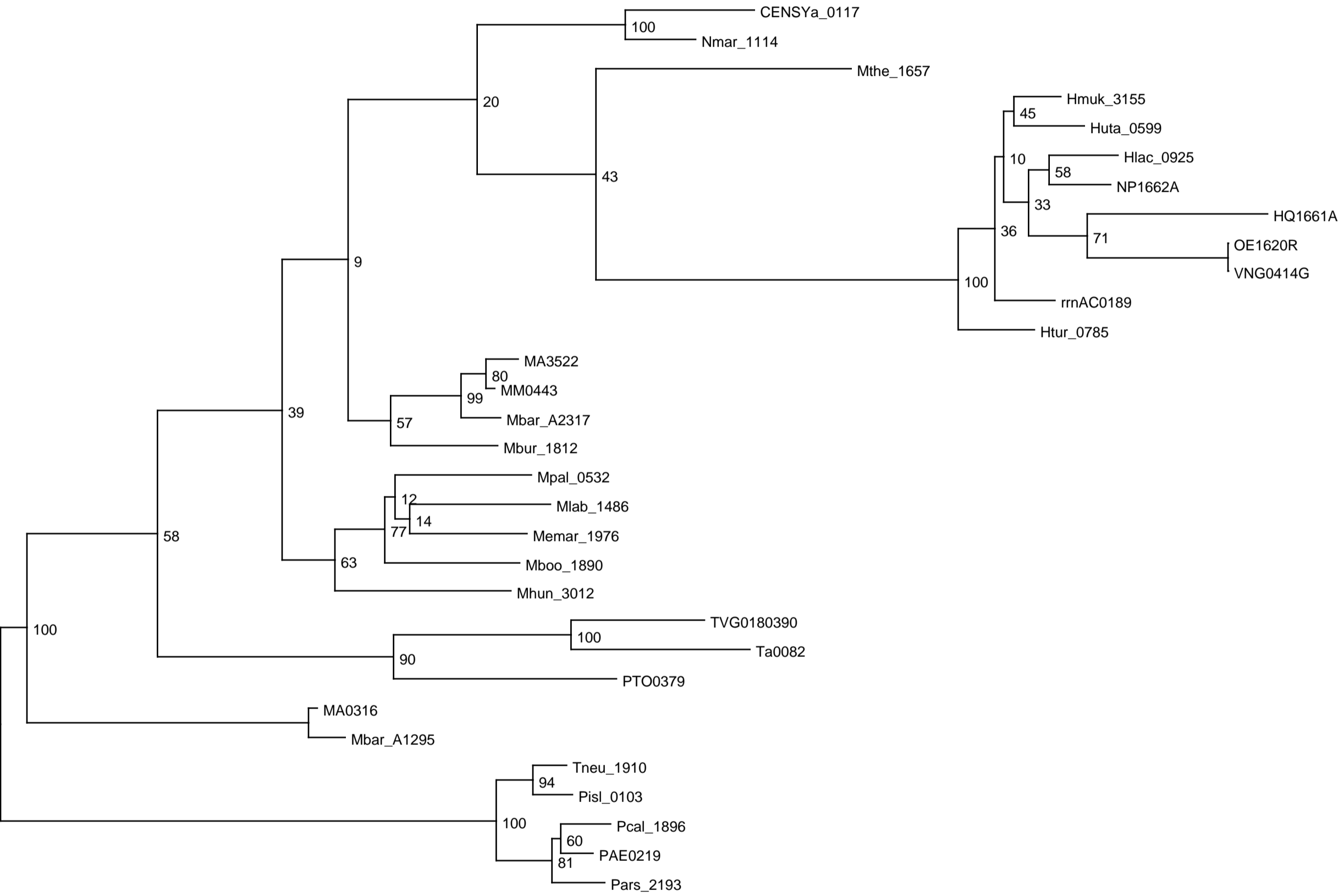

Supplement: Additional file 2 — Zip file containing additional phylogenetic trees. A set of phylogenetic trees generated as described in the Methods section. Locus tags were used for archaeal proteins, while species names were used for non-archaeal proteins used for comparisons. [file 1745-6150-6-63-S2.ZIP › Supplemental data file 1/PurN tree.pdf]

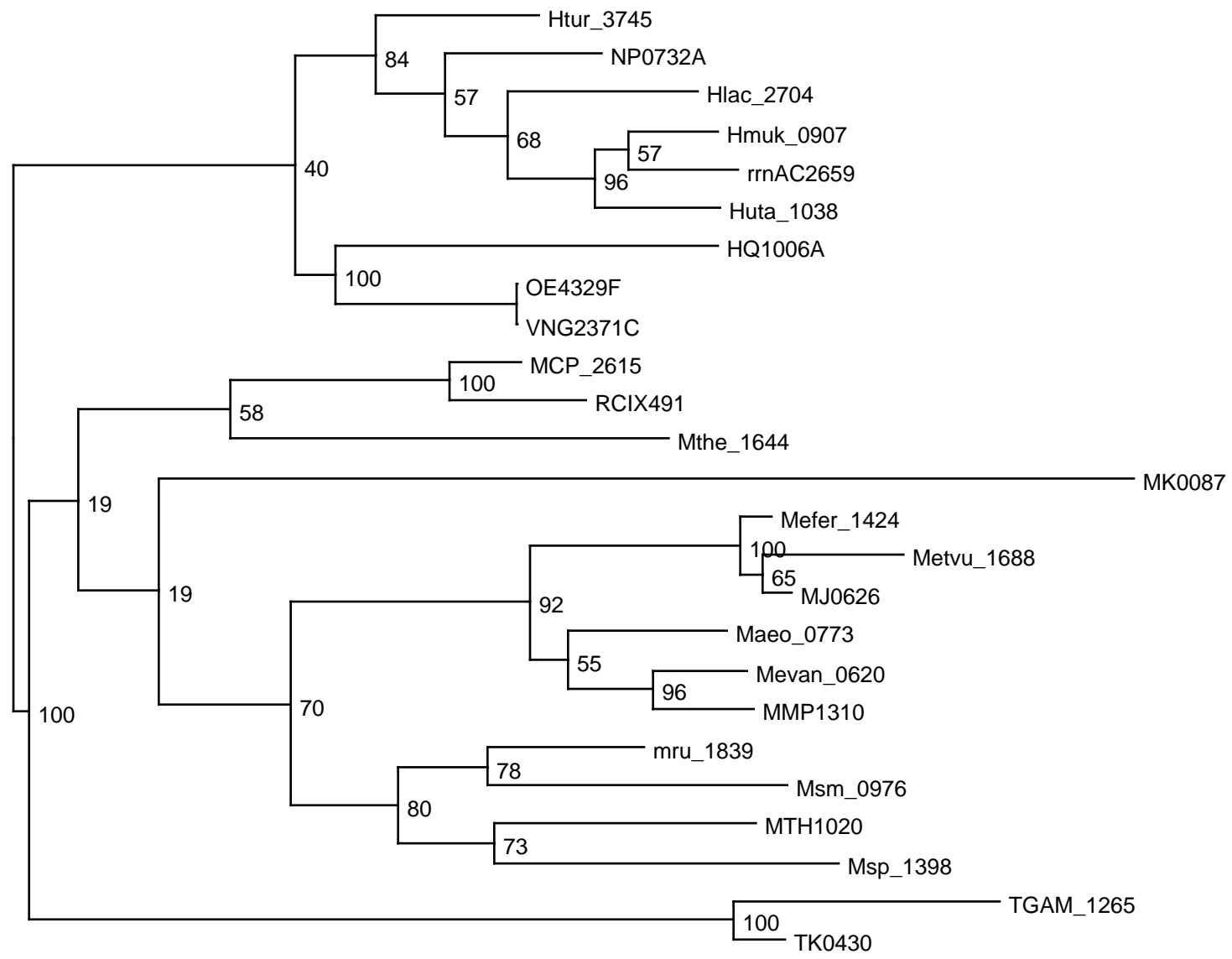

Supplement: Additional file 2 — Zip file containing additional phylogenetic trees. A set of phylogenetic trees generated as described in the Methods section. Locus tags were used for archaeal proteins, while species names were used for non-archaeal proteins used for comparisons. [file 1745-6150-6-63-S2.ZIP › Supplemental data file 1/PurO tree.pdf]

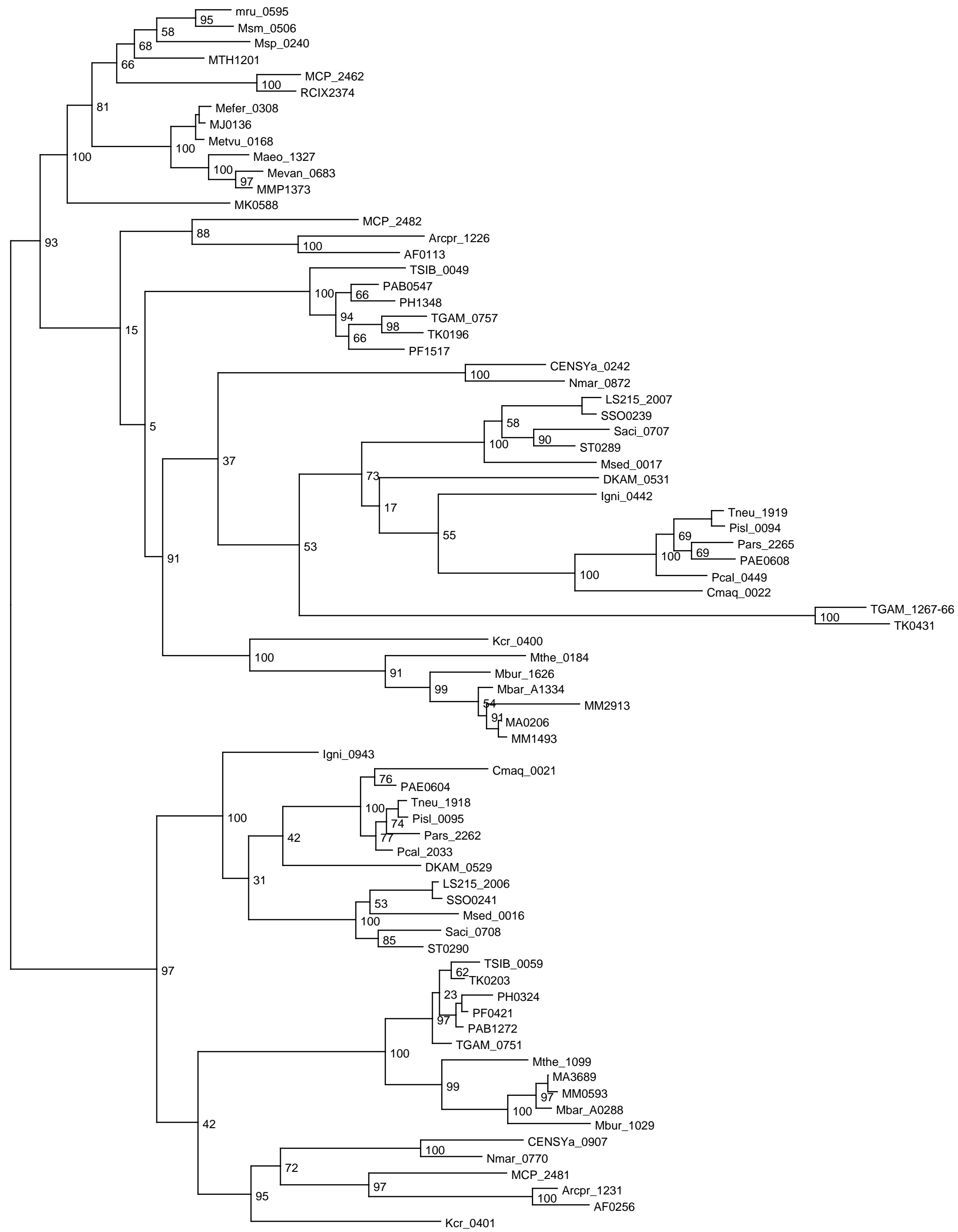

Supplement: Additional file 2 — Zip file containing additional phylogenetic trees. A set of phylogenetic trees generated as described in the Methods section. Locus tags were used for archaeal proteins, while species names were used for non-archaeal proteins used for comparisons. [file 1745-6150-6-63-S2.ZIP › Supplemental data file 1/PurP tree.pdf]

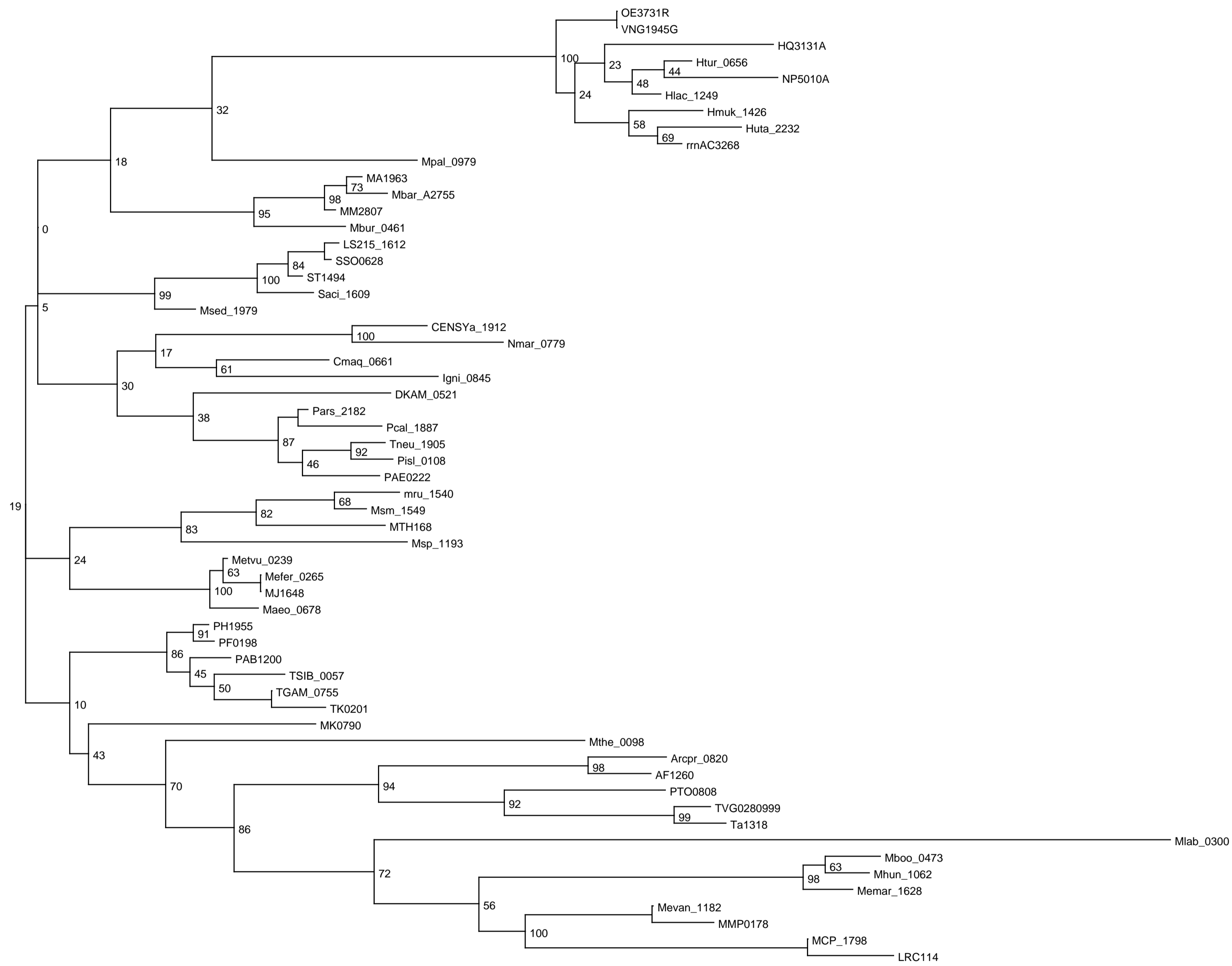

Supplement: Additional file 2 — Zip file containing additional phylogenetic trees. A set of phylogenetic trees generated as described in the Methods section. Locus tags were used for archaeal proteins, while species names were used for non-archaeal proteins used for comparisons. [file 1745-6150-6-63-S2.ZIP › Supplemental data file 1/PurQ tree.pdf]

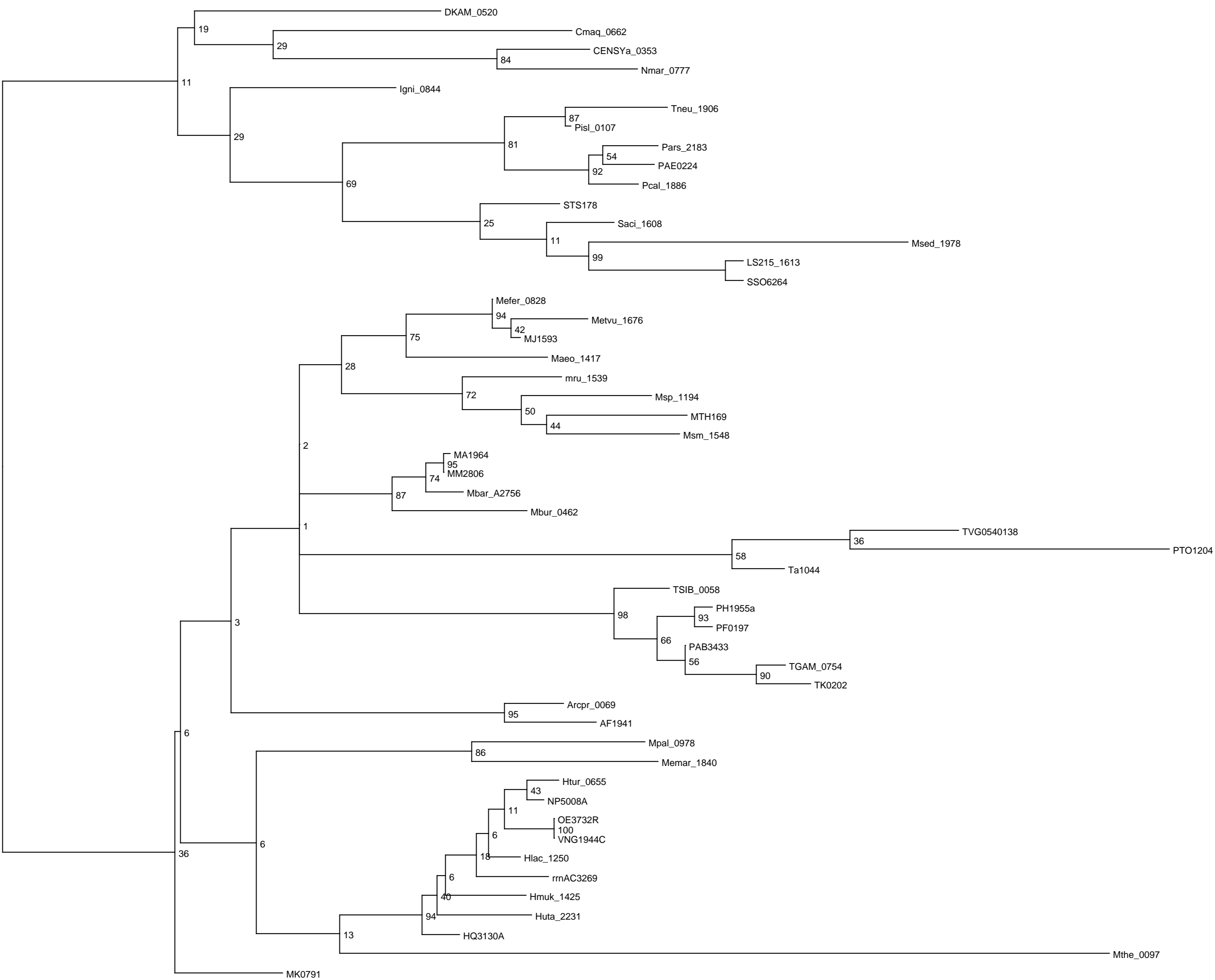

Supplement: Additional file 2 — Zip file containing additional phylogenetic trees. A set of phylogenetic trees generated as described in the Methods section. Locus tags were used for archaeal proteins, while species names were used for non-archaeal proteins used for comparisons. [file 1745-6150-6-63-S2.ZIP › Supplemental data file 1/PurS tree.pdf]

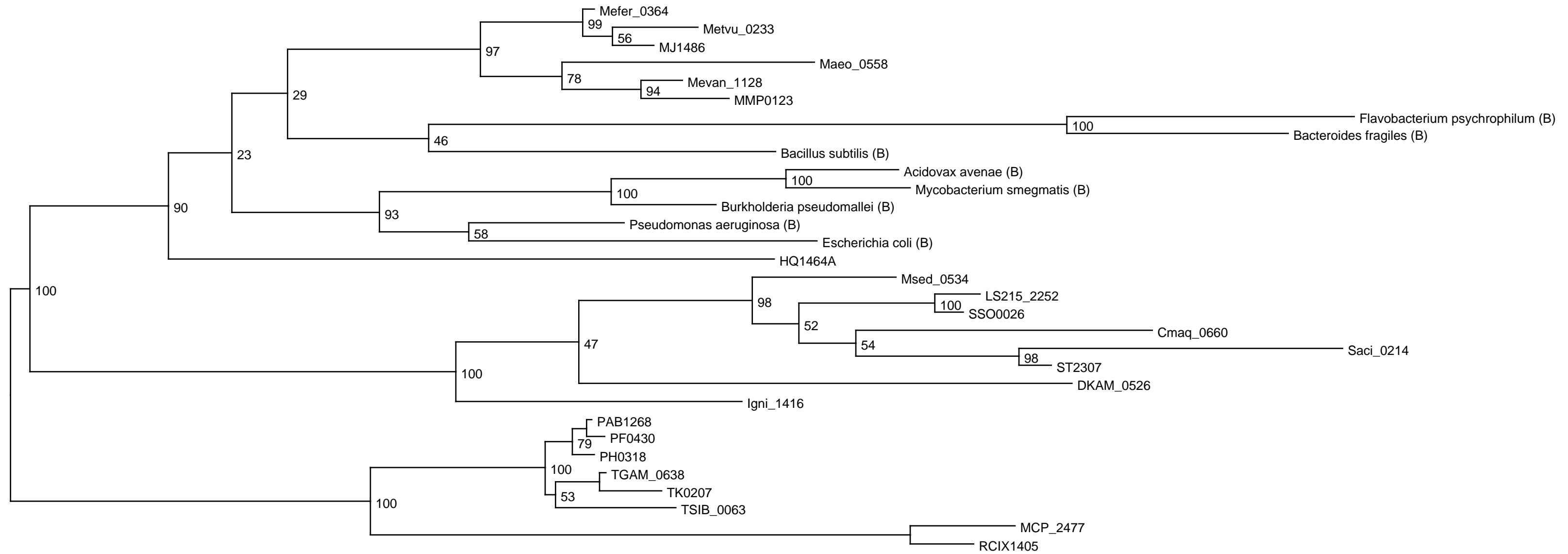

Supplement: Additional file 2 — Zip file containing additional phylogenetic trees. A set of phylogenetic trees generated as described in the Methods section. Locus tags were used for archaeal proteins, while species names were used for non-archaeal proteins used for comparisons. [file 1745-6150-6-63-S2.ZIP › Supplemental data file 1/PurT tree with non-archaea.pdf]
